# Dietary Butyrate Helps to Restore the Intestinal Status of a Marine Teleost (*Sparus aurata*) Fed Extreme Diets Low in Fish Meal and Fish Oil

**DOI:** 10.1371/journal.pone.0166564

**Published:** 2016-11-29

**Authors:** Itziar Estensoro, Gabriel Ballester-Lozano, Laura Benedito-Palos, Fabian Grammes, Juan Antonio Martos-Sitcha, Liv-Torunn Mydland, Josep Alvar Calduch-Giner, Juan Fuentes, Vasileios Karalazos, Álvaro Ortiz, Margareth Øverland, Ariadna Sitjà-Bobadilla, Jaume Pérez-Sánchez

**Affiliations:** 1 Fish Pathology Group, Instituto de Acuicultura Torre de la Sal (IATS-CSIC), Castellón, Spain; 2 Nutrigenomics and Fish Endocrinology Group, Instituto de Acuicultura Torre de la Sal (IATS-CSIC), Castellón, Spain; 3 Department of Animal and Aquacultural Sciences, Norwegian University of Life Sciences, Ass, Norway; 4 Comparative Endocrinology and Integrative Biology. CCMar, University of Algarve, Faro, Portugal; 5 BioMar R&D, Grangemouth, United Kingdom; 6 Norel S.A., Madrid, Spain; Universidade de Vigo, SPAIN

## Abstract

There is a constant need to find feed additives that improve health and nutrition of farmed fish and lessen the intestinal inflammation induced by plant-based ingredients. The objective of this study was to evaluate the effects of adding an organic acid salt to alleviate some of the detrimental effects of extreme plant-ingredient substitution of fish meal (FM) and fish oil (FO) in gilthead sea bream diet. Three experiments were conducted. In a first trial (T1), the best dose (0.4%) of sodium butyrate (BP-70 ^®^NOREL) was chosen after a short (9-weeks) feeding period. In a second longer trial (T2) (8 months), four diets were used: a control diet containing 25% FM (T2-D1) and three experimental diets containing 5% FM (T2-D2, T2-D3, T2-D4). FO was the only added oil in D1, while a blend of plant oils replaced 58% and 84% of FO in T2-D2, and T2-D3 and T2-D4, respectively. The latter was supplemented with 0.4% BP-70. In a third trial (T3), two groups of fish were fed for 12 and 38 months with D1, D3 and D4 diets of T2. The effects of dietary changes were studied using histochemical, immunohistochemical, molecular and electrophysiological tools. The extreme diet (T2-D3) modified significantly the transcriptomic profile, especially at the anterior intestine, up-regulating the expression of inflammatory markers, in coincidence with a higher presence of granulocytes and lymphocytes in the submucosa, and changing genes involved in antioxidant defences, epithelial permeability and mucus production. Trans-epithelial electrical resistance (Rt) was also decreased (T3-D3). Most of these modifications were returned to control values with the addition of BP-70. None of the experimental diets modified the staining pattern of *PCNA*, *FABP2* or *ALPI*. These results further confirm the potential of this additive to improve or reverse the detrimental effects of extreme fish diet formulations.

## Introduction

The availability of wild fishery-derived raw materials is finite and the rapid and sustained growth rate of global aquaculture have forced the industry to explore alternative and more sustainable feed ingredients [1, 2]. Much attention has been paid to plant ingredients and experimental evidence supports a successful replacement of marine feedstuffs at relatively high levels in many fish species, including salmonid and non-salmonid fish [3–6]. The positive impact of the early-feeding of a plant-based diet on its future acceptance and utilisation has also been reported [7]. However, low fish meal (FM) inclusion levels remain associated to poor growth and survival in different fish species [8–11]. Likewise, when low fish oil (FO) inclusion levels are considered, the nutritional value of farmed fish is compromised by a low content of ω-3 long-chain polyunsaturated fatty acids [12, 13]. Thus, a better understanding of the long term physiological consequences of plant-based diets or other alternative feed ingredients is a major issue to improve aquaculture sustainability. At the same time, there is an increasing interest for fish feed additives to prevent or repair adverse effects of extreme diet formulations, which might result on impaired growth, enteritis or immune-suppression [14–16].

One promising feed additive is butyrate, a salt of short chain-fatty acid (SCFA) produced by bacterial fermentation of undigested carbohydrates, which has received special attention for its positive effect on gut health in humans and livestock animals [17]. The potential to influence a wide array of cellular functions such as inhibition of inflammation and carcinogenesis, reinforcement of various components of the colonic defence barrier and reduction of oxidative stress have been reported in humans [18, 19]. Likewise, dietary butyrate improves the intestinal morphology and function of chicken and partially attenuates inflammatory responses caused by liposaccharide [20]. Potential benefits of butyrate have also been proved in pigs, and the reduction in diarrhoea incidence by the addition of butyrate has been attributed to the improvement of intestinal integrity [21].

In fish, dietary butyrate is able to improve growth and feed utilization in carp [22, 23], although no consistent effects have been reported in rainbow trout [24]. By contrast, in gilthead sea bream, dietary butyrate has been shown to provide energy for the epithelial intestine with a slight improvement of growth rates [25]. Recent data in European sea bass have shown an improvement of growth and increased gene expression of the oligopeptide transporter 1 in the hindgut of fish fed low FM diets supplemented with butyrate [26]. However, its addition at 0.2% in the same fish species did not produce any change in growth performance, but changes in the expression of genes related to mucosal protection and inflammatory response [27]. The underlying mechanisms of the action of butyrate remain unclear and poorly studied in fish. Accordingly, the main goal of the present study is to define if butyrate supplementation has the potential to exert benefits upon the intestine of gilthead sea bream. A first trial (T1) was performed to define the best-effective butyrate dose, mostly based on the effects on growth performance and hepatic and intestinal histopathology. In a second (T2) and third (T3) trial, the selected dose was added to an extreme low FM/FO diet to test the long-term effectiveness to prevent an inflammatory condition or other metabolic dysfunctions on the basis of different histochemical, immunohistochemical, electrophysiological and molecular features.

## Materials and Methods

### Diets and experimental design

**Fig 1:**
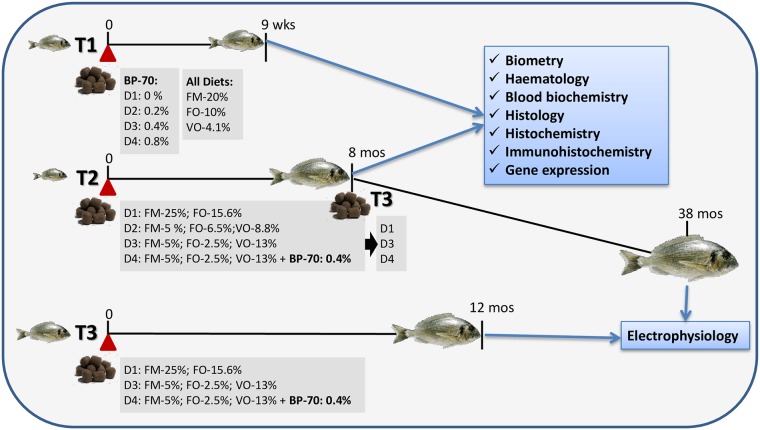
Diagrammatic summary of the different gilthead sea bream feeding trials (T) showing their timing, the main features of diet composition and the analyses performed at the end of each of them.

Juvenile gilthead sea bream of Atlantic origin (Ferme Marine de Douhet, Ile d’Oléron, France) were acclimatized for four weeks at the Institute of Aquaculture Torre de la Sal (IATS). Three different trials were performed, which are summarized in the diagram shown in Fig 1, for clarity. In a first trial (T1) fish, with an initial body weight of 26–27 g were randomly distributed in triplicated 90 L tanks (20 animals/tanks). Fish were fed for 9 weeks (August-October 2012) diets (Sparos Lda, Faro, Portugal) containing 20% fish meal (FM), 10% fish oil and graded levels of a commercial sodium butyrate preparation (BP-70 ^®^Norel) (0%, T1-D1 diet; 0.2%, T1-D2 diet; 0.4%, T1-D3 diet; 0.8%, T1-D4 diet) (S1 Table). Gustor BP-70 is a partially protected sodium butyrate, with 70% of sodium butyrate and 30% of fat. This small amount of fat allows the active principle to be active along the entire gastrointestinal tract.

In a second trial (T2), juvenile fish of 15–16 g initial body weight were allocated in 2500 L tanks in triplicated groups (150 fish/tank) and fed for 8 months (May-November 2013) four experimental diets formulated and produced by BioMar (Brande, Denmark), according to the experimental setup described in [28]. FM was added at 25% in the control diet (T2-D1) and at 5% in the other three diets (T2-D2, T2-D3, T2-D4). Added oil was either FO (T2-D1) or a blend of plant oils (VO) replacing 58% (T2-D2) and 84% (T2-D3, T2-D4) of the FO. The BP-70 compound was added to T2-D4 diet at 0.4%. All diets were isonitrogenous, isolipidic and isoenergetic and met all known nutritional requirements of gilthead sea bream. The composition of the experimental diets can be found in S2 Table.

In a third trial (T3), a subgroup of T2 fish was further fed D1, D3 and D4 diets (75 fish/diet group) for additional 30 months until reaching a mean weight of 1,420 g (T3-A), and another stock of juvenile fish was fed also D1, D3 and D4 diets (150 fish/diet group), all in triplicated groups for 12 months until reaching a mean weight of 249 g (T3-B). Both groups were used only for electrophysiological studies simultaneously in May 2016. Biometric data of T3 fish can be found in [29].

In all trials, oxygen content of outlet water remained higher than 85% saturation, and day-length and water temperature followed natural changes at IATS latitude (40° 5’N; 0° 10’E). Groups were fed to visual satiety by hand (until fish were not swimming actively towards the water surface to eat the pellets) once-twice per day, 3–6 days per week depending on season and fish size. Body weight was determined collectively every 3–6 weeks to evaluate the effects of experimental diets on specific growth rates. Feed conversion ratio was calculated [FCR = 100 x (dry feed intake / wet weight gain)].

### Sampling procedures and ethics statement

At the end of T1 and T2 trials and following overnight fasting, four randomly sampled fish from each dietary replicate (12–15 fish per dietary group) were anaesthetised with 3-aminobenzoic acid ethyl ester (100 mg/ml) and blood and tissue samples were obtained for haematological, histological and transcriptomic analyses. Blood was taken by puncture of caudal vessels with heparinized syringes. An aliquot was used to measure haemoglobin concentration and plasma samples of the remaining blood were stored at -20°C until analyses of glucose, cortisol and antioxidant activity.

Viscera, intestine and liver alone were dissected to calculate viscerosomatic index [VSI = 100 x (viscera weight/fish weight)], hepatosomatic index [HSI = 100 x (liver weight/fish weight)] and gut index [GI = 100 x (body weight/intestine length^3)]. Pieces of liver and three intestinal segments (anterior, AI; middle, MI; posterior, PI) were fixed in 10% buffered formalin for histochemical and immunohistochemical studies. For transcriptomic analyses, additional AI and PI samples were frozen in liquid nitrogen and stored at -80°C until.

All procedures were approved by the Ethics and Animal Welfare Committee of Institute of Aquaculture Torre de la Sal (Spain) and supervised by the Ethics experts of the TNA selection panel of the AQUAEXCEL and AQUAEXCEL^2020^ projects. It was carried out in a registered installation facility (code 36271-42-A) in accordance with the principles published in the European animal directive (2010/63/EU) and Spanish laws (Royal Decree RD53/2013) for the protection of animals used in scientific experiments. For lethal samplings, the suffering of animals was kept to a minimum.

### Blood biochemistry

Haemoglobin concentration was determined in fresh samples with a HemoCue B-Haemoglobin Analyser^®^ (AB, Leo Diagnostic, Sweden), which uses a modified azide methaemoglobin reaction. Briefly, blood was drawn into disposable microcuvettes which contain reagents in dried form that produce the red blood cell lysis and the conversion of haemoglobin to methaemoglobin by sodium nitrate, which is then combined with azide. The absorbance of the azide methaemoglobin is then photometrically measured at 565 nm and 880 nm. Plasma glucose levels were measured by the glucose oxidase method (ThermoFisher Scientific, Waltham, Massachusetts, USA) adapted to 96-well microplates. Briefly, this method is based on the oxidation of beta D-glucose present in the plasma to D gluco-1,5-lactone with the formation of hydrogen peroxide. Plasma total antioxidant capacity was determined with a commercial kit (Cayman Chemical, Ann Arbor, Michigan, USA) adapted to 96-well microplates, by measuring the inhibition of the oxidation of ABTS (2,2′-azino-di-[3-ethylbenzthiazoline sulphonate]) quantified as millimolar Trolox equivalents. Plasma cortisol levels were analysed using EIA kit (Arbor Assays) (detection limit 45 pg/mL) with intra- and inter-assay coefficients of variation lower than 3% and 5%, respectively.

### Histochemistry and immunohistochemistry

Fixed tissue samples (AI, MI, PI in T1 and AI, PI in T2) were dehydrated in graded ethanol series, embedded in paraffin, 4 μm-sectioned and stained with haematoxylin-eosin, giemsa or periodic acid-Schiff (PAS). Immunolabelling of *FABP2* and *PCNA* was carried out by incubating for one hour either with goat Pab anti-FABP2 (1:300) (Novus Biologicals, CO, USA) or with mouse Mab anti-PCNA (1:200) (Dako, Glostrup, Denmark). The corresponding biotinylated secondary antibodies (1:200) (VECTOR Labs.) (1h) and avidin-biotin-peroxidase complex (VECTOR Labs.) (30 min) were added. Bound peroxidase was visualized with 3,3’-diaminobenzidine tetrahydrochloride (Sigma, St. Louis, MO, USA) and sections were counterstained with Gill’s haematoxylin, dehydrated and mounted in DPX. *FABP2* antigens were previously retrieved by heating in a pressure-cooker for 15 min in 10 mM sodium citrate buffer (pH 6.0). *ALPI* activity was detected by incubating for 10 min with 5-bromo-4-chloro-3-indolyl phosphate/Nitro blue tetrazolium (Sigma), followed by washing in deionised water, dehydrating and mounting in DPX. The intensity of the staining for the three molecules and PAS was evaluated visually, in a blind mode, by a single observer according to a semiquantitative scale ranging from 1 (weak) to 3 (strong), based on the strength and extension of the staining. All the slides were stained in a single batch at the same time for each technique, to avoid staining differences due to the manipulation process.

### Gene expression

Total RNA was extracted using the MagMAX-96 total RNA isolation kit (Life Technologies, Madrid, Spain). The RNA yield was 50–100 μg and RIN (RNA integrity number) values were 8–10 with the Agilent 2100 Bioanalyzer. Reverse transcription (RT) of 500 ng total RNA was performed with random decamers, using the High-Capacity cDNA Archive Kit (Applied Biosystems, Madrid, Spain) following manufacturer’s instructions. Negative control reactions were run without reverse transcriptase and real-time quantitative PCR was carried out with a CFX96 Connect^™^ Real-Time PCR Detection System (Bio-Rad, Hercules, CA, USA). In samples from T1, the intestinal expression of three selected genes was analysed (*FABP2*, *PCNA*, *ALPI*). For samples of T2, a 96-well PCR array layout was used for the simultaneous profiling of 86 genes under uniform cycling conditions as reported elsewhere [30]. This set of genes included markers of cell differentiation and proliferation, intestinal architecture and permeability, enterocyte mass and epithelial damage, immune-surveillance, pattern recognition receptors (PRRs), and mitochondria function and biogenesis (Table 1). Controls of general PCR performance were included, and all the liquid manipulations were made with the EpMotion 5070 Liquid Handling Robot (Eppendorf, Hamburg, Germany). PCR-wells contained a 2x SYBR Green Master Mix (Bio-Rad, Hercules, CA, USA), and specific primers at a final concentration of 0.9 μM were used to obtain amplicons of 50–150 bp in length (S3 Table). PCR amplification included an initial denaturation step at 95°C for 3 min, followed by 40 cycles of denaturation for 15 s at 95°C and annealing/extension for 60 s at 60°C. The specificity of reactions was verified by analysis of melting curves and linearity of serial dilutions of RT reactions. Fluorescence data acquired during the PCR extension phase were normalised using the delta-delta Ct method [31] and β-actin was used as a housekeeping gene (mean CTs varied between 21.71 and 21.89).

**Table 1:** Gilthead sea bream genes included in real-time PCR by six functional categories. **1** Cell differentiation and proliferation; **2** Intestinal architecture and permeability; **3** Enterocyte mass and epithelial damage; **4** Immune surveillance; **5** Pattern recognition receptors; **6** Mitochondria function and biogenesis.

Gene name/category	Symbol	Gene name/category	Symbol
*Category 1*		*Category 2*	
Proliferating cell nuclear antigen	*PCNA*	Integrin beta-1-binding protein 1	*ITGB1BP1*
Bone morphogenetic protein receptor type-1A	*BMPR1A*	Integrin beta-6	*ITGB6*
Indian hedgehog protein	*IHH*	Integrin-linked protein kinase	*ILK*
Zinc finger protein GLI1	*GLI1*	Occludin	*OCLN*
Hedgehog-interacting protein	*HHIP*	Claudin 12	*CLDN12*
Protein wntless homolog	*WLs*	Claudin 15	*CLDN15*
Transcriptional regulator Myc	*Myc*	Tight junction protein ZO-1	*TJP1*
Catenin beta-1	*CTNNB1*	Cadherin 1	*CDH1*
Transcription factor 4	*Tcf4*	Cadherin 17	*CDH17*
Notcheless protein homolog 1	*NLE1*	Junctional adhesion molecule A	*F11R*
Transcription factor HES-1-B	*HES1-B*	Coxsackievirus and adenovirus receptor homolog	*CXADR*
Zinc finger protein GFI-1	*GFI-1*	Desmoplakin	*DSP*
Krueppel-like factor 4	*KLF4*	Gap junction Cx32.2 protein	*CX32*.*2*
		Gap junction beta-4 protein	*GJB4*
		Mucin 2	*MUC2*
		Mucin 2-like	*MUC2-like*
		Mucin 13	*MUC13*
		Intestinal mucin	*I-MUC*
*Category 3*		*Category 4*	
Intestinal-type alkaline phosphatase	*ALPI*	Interleukin 1 beta	*IL-1β*
Liver type fatty acid-binding protein	*FABP1*	Interleukin 1 receptor type 1	*IL-1R1*
Intestinal fatty acid-binding protein	*FABP2*	Interleukin 6	*IL-6*
Ileal fatty acid-binding protein	*FABP6*	Interleukin 6 receptor subunit beta	*IL-6RB*
Calreticulin	*CALR*	Interleukin 7	*IL-7*
Calnexin	*CANX*	Interleukin 8	*IL-8*
Glutathione reductase	*GR*	High affinity interleukin-8 receptor A	*IL-8RA*
Glutathione S-transferase 3	*GST3*	Interleukin 10	*IL-10*
Superoxide dismutase [Cu-Zn], cytoplasmatic	*SOD1*	Interleukin10 receptor subunit alpha	*IL-10RA*
Peroxiredoxin 1	*PRDX1*	Interleukin 12 B	*IL-12B*
Peroxiredoxin 2	*PRDX2*	Tumor necrosis factor alpha	*TNFα*
		Macrophage colony-stimulating factor 1 receptor 1	*CSF1R1*
		C-X-C motif chemokine	*CXC*
		C-C chemokine receptor type 3	*CCR3*
		C-C chemokine receptor type 9	*CCR9*
		C-C chemokine receptor type 11	*CCR11*
		C-C chemokine CK8	*CK8*
		CD48 antigen	*CD48*
		CD276 antigen	*CD276*
*Category 5*		*Category 6*	
Toll-like receptor 1	*TLR1*	Mitochondrial 10 kDa heat shock protein	*mtHsp10*
Toll-like receptor 2	*TLR2*	Mitochondrial 60 kDa heat shock protein	*mtHsp60*
Toll-like receptor 5	*TLR5*	Mitochondrial 70 kDa heat shock protein	*mtHsp70*
Toll-like receptor 9	*TLR9*	Enoyl-CoA hydratase	*ECH*
Nucleotide-binding protein oligomerization domain-containing protein 1	*NOD1*	Hydroxyacyl-CoA dehydrogenase	*HADH*
Macrophage mannose receptor 1	*MRC1*	Citrate synthase	*CS*
CD209 antigen	*CD209*	Mitochondrial import inner membrane translocase subunit 44	*Tim44*
CD302 antigen	*CD302*	Mitochondrial import receptor subunit Tom22	*Tom22*
C-type lectin domain family 10 member A	*CLEC10A*	Mitochondrial transcription factor A	*mtTFA*
Galectin-1	*LGALS1*	Nuclear respiratory factor 1	*NRF1*
Galectin-8	*LGALS8*	Proliferator-activated receptor gamma coactivator 1 alpha	*PGC1α*
L-rhamnose-binding lectin CSL2	*CSL2*		
Fucolectin	*FCL*		
Vimentin	*VIM*		

### Ussing chamber assays for electrophysiology

The anterior intestine of anaesthetized fish from T3 (10–12 fish/diet group/size) was collected, isolated and mounted in Ussing chambers as previously described [32, 33]. Briefly, AI was washed with chilled physiological saline, opened flat, placed on a tissue holder of 0.71 cm^2^ and positioned between two half- chambers containing 2 mL of physiological saline at pH 7.80. During the experiments the tissue was bilaterally gassed with 0.3% CO_2_ + 99.7 O_2_ and kept at 17°C. Epithelia were clamped to 0 mV and trans-epithelial electrical resistance (Rt, Ω cm^2^), a measure of tissue integrity, was manually calculated (Ohm’s law) using the current deflections induced by a 2 mV pulse of 3 s every minute. Voltage clamping and current injections were performed with VCC600 or VCCMC2 amplifiers (Physiologic Instruments, San Diego, USA). Bioelectrical parameters for each tissue were manually recorded at 30 min intervals from 30–150 min after mounting.

### Statistical analysis

Data on gene expression, Ussing chamber assays and staining intensity in histological sections were analysed using one-way analysis of variance (ANOVA-I), followed by a Student-Newman-Keuls post hoc test. When the test of normality or equal variance failed, a Mann-Whitney Rank Sum test or a Kruskal-Wallis ANOVA on ranks followed by Dunn’s method was applied instead, respectively. For the staining intensity, when no differences among diets were found, data were merged and differences between the two intestinal segments were analysed *via* Student's t-test. The significance level was set at *P* < 0.05. All analyses were conducted using SPSS package version 19.0 (SPSS Inc., Chicago, IL, USA).

## Results

### Butyrate effects on growth performance

Fish in T1 grew efficiently from 26–27 g to 74–79 g with similar SGR (1.71–1.74) and FCR (1.1–1.2), regardless of the butyrate supplementation level (Table 2). The dietary treatment did not alter significantly VSI and HSI, but the GI was significantly higher in fish fed the diet with the highest butyrate dose (T1-D4) than in the remaining fish groups. Blood haemoglobin concentration and plasma total antioxidant capacity were not significantly altered. However, plasma glucose increased with butyrate supplementation with a maximum in fish fed the intermediate level (0.4%, T1-D3), which was significantly different from values found in the control group. The same pattern was found for plasma cortisol, although the trend was not statistically significant.

**Table 2:** Dose dependent effects of sodium butyrate (BP-70 ^®^Norel) on growth performance and blood parameters of gilthead sea ream in trial 1 (T1). Fish were fed four diets: T1-D1 (control), T1-D2 (0.2% BP-70), T1-D3 (0.4% BP-70) and T1-D4 (0.8% BP-70). Data on body weight, feed intake, and growth indices are the mean ± SEM of triplicate tanks. Data on viscera and liver weight are the mean ± SEM of 24 fish. Data on plasma parameters are the mean ± SEM of 12 fish. Different superscript letters in each row indicate significant differences among dietary treatments (*P* < 0.05; Student-Newman-Keuls).

	T1-D1	T1-D2	T1-D3	T1-D4	*P-value*
Initial body weight (g)	26.6±0.14	26.6±0.09	26.6±0.41	26.8±0.45	*0*.*932*
Final body weight (g)	74.6±2.30	73.8±2.01	78.0±2.14	78.7±2.39	*0*.*513*
Feed intake (g DM/fish)	54.7±1.98	51.7±1.63	55.1±1.81	58.5±2.10	*0*.*167*
Viscera weight (g)	4.99±0.20^a^	4.96±0.20^a^	5.14±0.18^a^	5.64±0.19^b^	*0*.*027*
Liver weight (g)	0.97±0.04	1.01±0.04	1.02±0.04	1.09±0.03	*0*.*155*
Intestine length (cm)	10.1±0.41	10.0±0.89	9.58±0.31	9.10±0.54	*0*.*180*
VSI (%)^1^	6.69±0.15	6.59±0.17	6.68±0.13	7.22±0.22	*0*.*064*
HSI (%)^2^	1.30±0.03	1.37±0.05	1.31±0.05	1.40±0.04	*0*.*161*
GI^3^	8.16±1.03^a^	9.17±1.79^a^	9.47±0.94^a^	13.2±1.55^b^	*0*.*050*
SGR (%)^4^	1.74±0.06	1.71±0.02	1.73±0.02	1.72±0.01	*0*.*918*
FCR (%)^5^	1.09±0.04	1.07±0.01	1.12±0.05	1.19±0.05	*0*.*320*
*Plasma parameters*					
Haemoglobin (g/dl)	9.23±0.20	9.17±0.16	9.36±0.34	9.27±0.30	*0*.*997*
Glucose (mg/dl)	46.4±1.7^a^	49.9±1.56^ab^	53.7±2.09^b^	51.3±1.10^ab^	*0*.*024*
Cortisol (ng/ml)	5.70±1.51	5.70±0.44	6.43±0.87	6.68±0.94	*0*.*364*
Antioxidant activity (Trolox mM)	1.28±0.06	1.06±0.12	1.17±0.06	1.08±0.06	*0*.*132*

^1^Viscerosomatic index = 100 x (viscera weight / fish weight)

^2^Hepatosomatic index = 100 x (liver weight / fish weight)

^3^Gut index = 100 x (fish weight / intestine length^3^)

^4^Specific growth rate = 100 x (ln final body weight—ln initial body weight) / days

^5^Feed conversion ratio = 100 x (dry feed intake / wet weight gain)

Data on growth performance in T2 are shown in detail in [28]. Briefly, all fish in T2 grew efficiently from 15 g to 296–320 g with SGR of 1.42–1.37% and FCR of 1–1.05. Hence, no detrimental effects on growth performance were found with the maximized and progressive replacement of FM and FO in T2-D2, T2-D3 and T2-D4 diets.

### Histological observations

**Fig 2:**
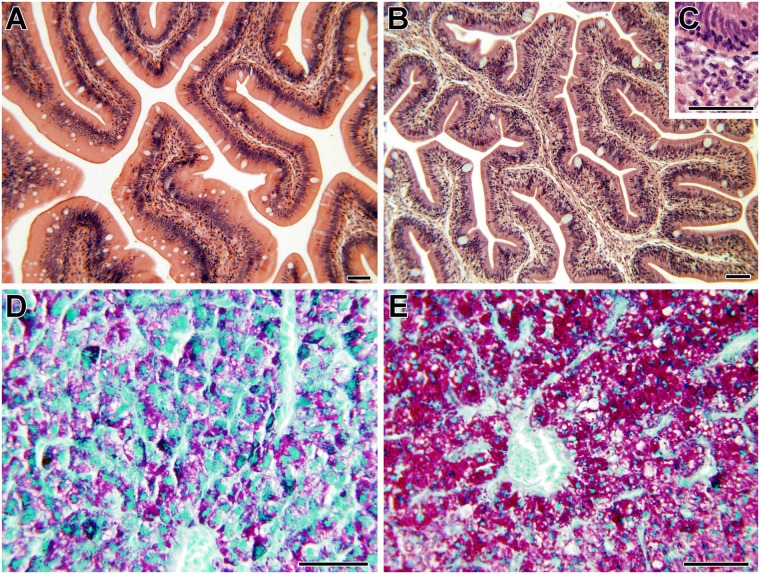
Effect of sodium butyrate (BP-70 ^®^Norel) on gilthead sea bream histological traits in trial 1 (T1). A, D: Representative images of fish fed the control diet without BP-70 (T1-D1); B, C and E: fish fed the diet with 0.8% BP-70 supplementation (T1-D4). Note the lymphocytic infiltration in the epithelial base and the eosinophilic granular cell abundance in insert C. Stainings = haematoxylin-eosin in posterior (A) and anterior (B, C) intestine (A, B, C) and periodic acid-Schiff in liver (D, E). Scale bars = 50 μm.

In T1, no strong differences along AI, MI and PI segments were detected. Only in fish fed T1-D4 an increase in the abundance of the mucosal foldings, a higher lymphocytic infiltration in the epithelial base, and a higher amount of granulocytes in the submucosa along the intestine was observed, as well as a remarkable increase in glycogen accumulation in hepatocytes revealed by PAS staining (Fig 2D and 2E).

In T2, few significant differences were found due to the diet. AI and PI segments from all the experimental diets showed abundant lymphocyte-like cells infiltrated at the epithelial base, compared to fish fed control diet (S1 Fig). Eosinophilic granule cells formed outstanding clusters in the lamina propria-submucosa of AI and PI of T2-D3 fish. PAS staining of goblet cells was significantly higher (*P* < 0.01) in AI than PI. Goblet cells were not stained with giemsa in the intestinal segments of most fish fed the control diet (T2-D1), whereas they were magenta stained in most fish fed the three experimental diets. In addition, goblet cells were especially abundant in T2-D4. T2-D4 livers showed a higher fat content in hepatocytes, a slightly higher amount of glycogen and a reduction of cord-like structures between islets of hepatocytes, without increasing glycogen content (S1 Fig).

### FABP2/PCNA immunodetection and ALPI activity

No significant differences were detected among the diet groups in any of the trials for immunohistochemical labelling of *FABP2*, *PCNA* or *ALPI* activity. However, segment differences were observed. *FABP2* cytoplasmic staining of the epithelial layer was typically stronger at AI than at PI, as clearly observed in T1 (S2A and S2B Fig), whereas in T2, 17% of the analysed fish had a patchy uneven immunolabelling at PI, mostly in T2-D3 fish. *PCNA* staining was predominantly found in the nuclei of epithelial cells, mainly enterocytes, though some *PCNA*^+^ cells were also detected in the lamina propria-submucosa. *PCNA*^+^ epithelial cells were mostly present in the intestinal intervillus pockets with decreasing density towards the apex of mucosal foldings and they were more abundant at AI than at PI in both trials, though segment differences were not significant (S2C and S2D Fig). *ALPI* activity was limited to the enterocyte brush border and the intensity of staining was significantly stronger at AI than at PI for samples of both trials (*P* < 0.001) (S2E and S2F Fig).

### Transcriptomic profiling

When the two extreme groups of T1 were analysed, significant differences (*P* < 0.05) were found for *PCNA* and *ALPI* through the intestinal tract. For both genes, constitutive expression at AI was significantly higher than at PI in T1-D1 and T1-D4 fish. However, a significant diet effect was only detected at AI, where a down-regulation of *PCNA* in fish fed the highest butyrate dose (T1-D4) was found (Table 3).

**Table 3:** Gene expression of the anterior and posterior intestine segments in gilthead sea bream fed the control diet (T1-D1) and the 0.8% sodium butyrate (BP-70 ^®^Norel) supplemented diet (T1-D4). Values are the mean ± SEM (n = 8). Different superscript letters in the same row indicate significant differences (P < 0.05; Student-Newman-Keuls).

Gene	Anterior intestine		Posterior intestine	
	T1-D1	T1-D4	T1-D1	T1-D4
*PCNA*^1^	1.96±0.23^a^	1.32±0.11^b^	0.89±0.10^c^	1.02±0.17^c^
*FABP2*^2^	82.75±18.74^a^	98.3±39.5^a^	0.26±0.11^b^	0.25±0.70^b^
*ALPI*^3^	53.7±4.76^a^	60.4±7.19^a^	12.1±1.23^b^	10.3±1.03^b^
*ILK*^4^	0.92±0.09^a^	0.95±0.08^a^	1.67±0.11^b^	1.47±0.12^b^

^1^Proliferating Cell Nuclear Antigen

^2^Intestinal Fatty Acid Binding Protein

^3^Intestinal Alkaline Phosphatase

^4^Integrin-linked kinase integrin-linked kinase

**Fig 3:**
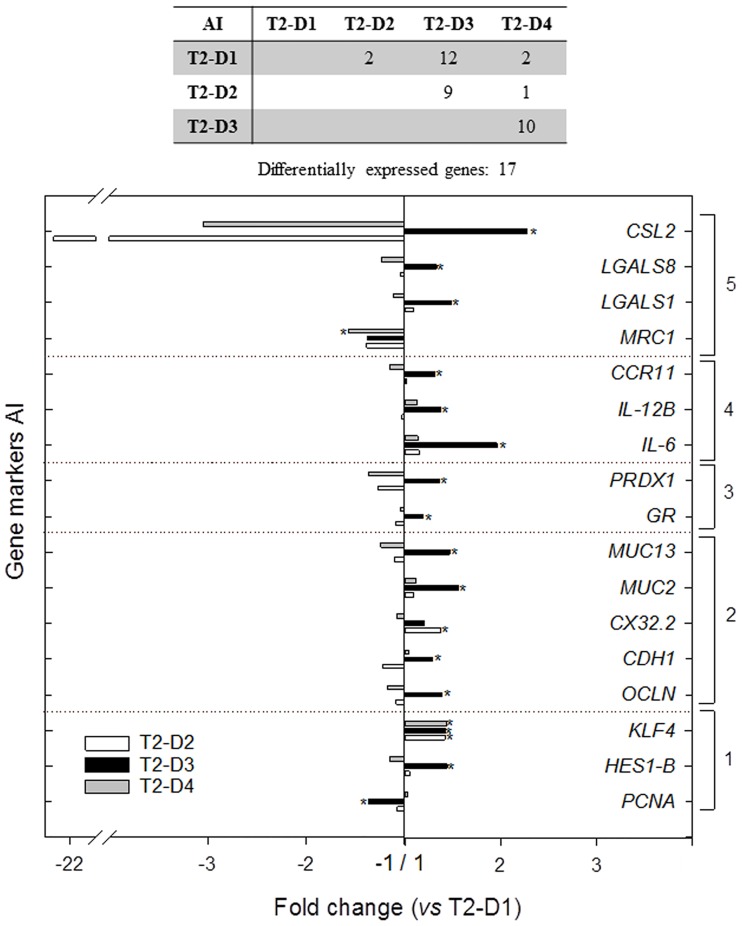
Differentially expressed genes at the anterior intestine (AI) of gilthead sea bream fed the three experimental substitution diets in trial 2 (T2). Fold changes are related to the control diet T2-D1. Bars above 1 stand for up-regulated genes and bars below 1 for down-regulated ones. Gene markers are grouped into 5 functional categories: 1 = cell differentiation and proliferation; 2 = intestinal architecture and permeability; 3 = enterocyte mass and epithelial damage; 4 = interleukins and cytokines; 5 = pattern recognition receptors. (*P < 0.05).

In T2, pooled samples from each dietary condition and intestine segment were first analysed to select the most responsive genes (up to 42 for each intestine segment) to dietary treatment, which were then targeted for gene expression profiling on samples from individual fish (n = 8). For AI, data on relative expression for this sub-set of genes is shown in S4 Table. At this intestinal segment, significant differences (*P* < 0.05) in the relative expression of 17 genes (40.5% of the analysed genes) were found among the different T2 diets. Fold changes of the significantly expressed genes *vs* T2-D1 are represented in Fig 3. Remarkable is that differentially expressed genes at the AI were found mainly in T2-D3 fed fish, (except for *CX32*.*2* and *MRC1*), and almost all of them were up-regulated (except *PCNA*). In particular, up-regulated genes at the AI of T2-D3 were related to cell differentiation and proliferation (*HES1-B* and *KLF4*), to intestinal architecture and permeability (*OCLN*, *CDH1*, *MUC2* and *MUC13*), to epithelial damage (*GR* and *PRDX1*) and to immunity (*IL6*, *IL12B*, *CCR11* and *LGALS1*, *LGALS8*, *CSL2*). No significant differences were detected between AI of T2-D2 and AI of T2-D4 gene expression (except for *CX32*.*2*), being the expression profiles at this intestinal segment of fish fed these two diets very similar to the expression profile of T2-D1.

**Fig 4:**
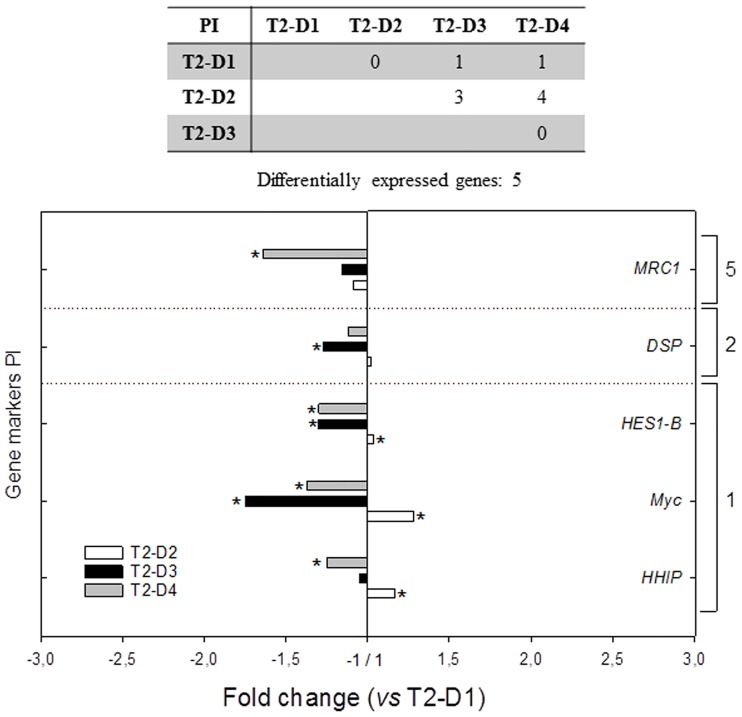
Differentially expressed genes at the posterior intestine (PI) of gilthead sea bream fed the three experimental substitution diets in trial 2 (T2). Fold changes are related to the control diet T2-D1. Bars above 1 stand for up-regulated genes and bars below 1 for down-regulated ones. Gene markers correspond to three functional categories: 1 = cell differentiation and proliferation; 2 = intestinal architecture and permeability; 5 = pattern recognition receptors. (*P < 0.05).

At the PI of T2 fish, the data on gene expression are shown in S5 Table. Significant transcriptional changes (*P* < 0.05) induced by the T2 experimental diets at PI were limited to five genes (11.9% of the analysed genes). Compared to AI, the significant gene regulation appeared to be of lower intensity at the PI, and an opposite trend of down-regulation was found in this intestinal segment. Fold changes of the significantly expressed genes *vs* T2-D1 are represented in Fig 4. Differential expression at PI occurred in fish fed both extremely substituted diets (T2-D3 and T2-D4) in genes related to cell differentiation and proliferation (*HHIP*, *Myc* and *HES1-B*), to intestinal architecture and permeability (*DSP*) and to PRRs (*MRC1*).

### Electrophysiology

**Fig 5:**
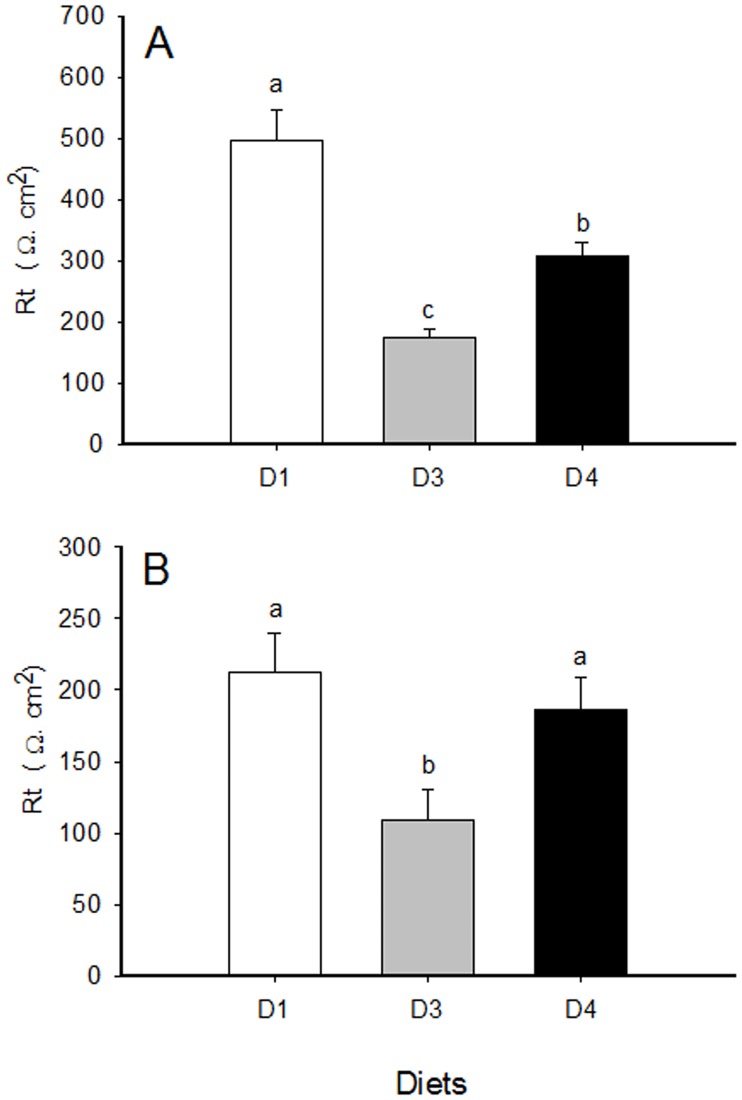
Trans-epithelial electrical resistance (Rt) of the anterior intestine of gilthead sea bream from trial 3 (T3). A: fish from T3-A (mean weight 1,420 g): B: fish from T3-B (mean weight 249 g)**.** Data are given as the mean ± SEM of the tissue resistance along the 120 min of *in vitro* experiments with Ussing chamber. Groups displaying different letters are significantly different (*P* < 0.05).

Rt values for each 30 min-point along the monitored period are shown in S3 Fig, and mean values for the 120 min-period of each AI are shown in Fig 5. Large fish (T3-A) showed higher Rt values than small ones (T3-B), regardless of the diet group. Dietary treatment induced modifications of the epithelial resistance in both T3 groups, as D3 fish (extreme diets) had significantly lower tissue Rt than fish fed control diet (D1). The decrease was higher in large fish fed D3 for 38 months than in small fish fed D3 for 12 months (~3.5-fold decrease *vs* ~1.9-fold). Butyrate supplementation (D4) improved tissue integrity, resulting in intermediate Rt values in both size groups, though only reaching D1 values in T3-B.

## Discussion

Previous studies have shown that inclusion of different feed additives, such as a bacterial meal (BM), its cell wall fraction, or *Candida utilis* and *Chlorella vulgaris*, can prevent the development of soy bean meal (SBM) induced enteritis in salmon [34–36]. In the present study, we have explored the possible beneficial effects of adding BP-70 to the diet of gilthead sea bream. The effects of butyric acid and butyrate in fish are not abundant and somehow contradictory. This is partly due to differences in the feeding protocols (duration of trials, fish age/size, diet formulations, products, dosage, etc.) [22–25, 27]. For this reason, the level of BP-70 was assessed in a first short trial (T1) which showed that most growth performance and blood parameters were not significantly changed by the three tested doses. The highest dose (0.8%, T1-D4) was rejected because it produced a mild inflammatory reaction in the intestine, and a pronounced glycogen accumulation in liver as a consequence of the increased glucose availability and transport. In rodents, glycogen hepatic storage was also a consequence of acute butyrate supply, due to the competition between butyrate and glucose for substrate oxidation [37]. Considering all this, T1-D3 dose (0.4% BP-70) was selected for the subsequent long-term feeding trial (T2) because fish did not show histopathological signs or reduced performance, and this dose was expected to have more significant effects in the long trial than the 0.2% dose (T1-D2).

It was previously shown that none of the experimental diets used in T2 affected growth performance, but the decrease in plasma haemoglobin and total cholesterol produced by the low inclusion levels of FM was reversed by butyrate [28]. In the current study, BP-70 (T2-D4) added to the extreme plant diet (T2-D3) reversed most of the detected detrimental intestinal signs. This was especially observed at AI, which was more responsive to the dietary treatments. In total, 17 genes were differentially expressed at AI *vs* only five at PI. T2-D3 fish in particular had the strongest regulation. This antero-posterior decreasing gradient could be explained, at least in part, by the fact that the delivery of the highest dose of BP-70, even being partially protected, is supposed to occur preferentially at the anterior intestine segment.

Most genes whose expression was modulated at AI are related to immune functions (cytokines and PRRs). The up-regulation of *IL-12ß*, *IL-6* and *CCR11* in T2-D3 fish reflected a pro-inflammatory status. Similarly, *IL-12ß* was up-regulated in the intestine of fish after pathogen exposure [38,39], and *IL-6* was significantly down-regulated in gilthead sea bream with a dietary-induced anti-inflammatory/anti-proliferative transcriptomic profile [30]. Inflammatory markers were also up-regulated in SBM-induced enteropathy in the distal intestine of Atlantic salmon [40], and the prevention of SBM-induced enteritis by BM was related to intestinal levels of *MHCII*- and *CD8α*-reactive cells [36]. These results agree with previous analysis in the same groups of fish, in which butyrate supplementation reversed the up-regulated expression of inflammatory cytokines (*TNFα*) and muscle markers of cellular morphogenesis and protein breakdown in the muscle of fish fed T2-D3. However, at hepatic level, the transcriptional-mediated changes in molecular chaperones, oxidative enzymes, immune-relevant genes, and master regulators of lipid metabolism were not returned to control values by butyrate [28]. Noticeably, no changes were found in T2-D2, indicating that T2-D3 response is probably related to the role of essential fatty acids (EPA and DHA) rather than to effects of FM replacement with plant raw materials.

Accordingly to this pro-inflammatory cytokine repertoire, the expression of PRR genes was also up-regulated (*CSL2*, *LGALS1*, *LGALS8*) in T2-D3 fish, but not in T2-D4 and T2-D2 fish. Galectins (*LGALS*) are part of the mucosal defence system in teleosts and *CSLs* are rhamnose-binding lectins that modulate innate immune responses by stimulating pro-inflammatory cytokine synthesis [41]. These fish PRRs exert differential functions and responses depending on the tissue, species, offending factor [42] and nutritional background [43]. In fact, the increased expression of other PRRs (*TLR*s, *NLRs)* has also been documented in salmon enteritis [34]. Furthermore, *MRC1*, a PRR for mannose including C-type lectin domains, was the only immune-related gene that was significantly down-regulated in T2-D4 fish. *MRC1* is involved in the synthesis and release of pro-inflammatory cytokines as well as in antigen acquisition and processing in mammals [44], but little is known about its role in fish [45]. Therefore, the observed down-regulation in T2-D4 fish would be consistent with the anti-inflammatory profile induced by BP-70. All this transcriptomic profile was consistent with the inflammatory histological signs detected along the intestine of fish fed T2-D3, with abundant eosinophilic granular cells and lymphocytic infiltrates.

Other genes up-regulated in the AI of T2-D3 fish were related to antioxidant defences (*PRDX1* and *GR*) and epithelial permeability and structure (tight-junction and adherens-junction proteins), probably entailing nutritional, osmoregulatory and physiological alterations. Similarly, increased intestinal permeability [46] and marked significant down-regulation of cell adhesion molecules were detected in salmon enteritis [34]. By contrast, the expression of genes involved in epithelial damage and intestinal architecture and permeability did not vary significantly in T2-D4 and T2-D2 in AI, suggesting again that the these effects are probably related to the extreme FO replacement with VO. The present results are in accordance with studies in human models that concluded that butyrate enhances the intestinal barrier by regulating the assembly of tight junctions [47], and beneficially affects oxidative stress [48].

T2-D3 diet also up-regulated two mucin genes (*MUC13* and *MUC2*) and two transcription factors (*KLF4* and *HES1-ß*) involved in goblet cell differentiation. The secretory *vs* absorptive fate of the continuously regenerated epithelial layer is regulated by these two factors, so that weak inhibitory *HES1* and strong stimulatory *KLF4* signals are required for goblet cell differentiation [49]. Accordingly, goblet cells appeared especially abundant in T2-D4 intestine.

No significant effects of the experimental diets on the staining pattern for *FABP2*, *ALPI* or *PCNA* were observed in both trials. The only observed differences were related to the intestinal segment. *FABP2* staining was clearly higher at AI than PI. This is in accordance with the significantly higher gene expression levels at AI in T1 and previous observations in fish [30, 50, 51], which is related to the major absorption of nutrients at AI. *ALP* has been extensively documented to be modulated by dietary intervention in mammalian models [52]. In fish, a general decline in the activity of *ALP* is associated to malnutrition, such as removal of FO in rainbow trout [53] or SBM induced enteritis in salmon [54]. However, *ALPI* transcripts were significantly down-regulated at AI in gilthead sea bream fed a prebiotic and NE150 combination [30] and intestinal *ALP* activity was not affected when meagre was fed a blend of VOs instead of FO [55]. By contrast, it was increased in European sea bass fed a blend of VOs replacing 70% FO [56]. Interestingly, in human colonic cells, butyrate induced *ALP* activity, but did not increase the transcripts of tissue-nonspecific *ALP*s [57]. The lack of changes in *ALPI* activity and expression in the current trials could indicate that none of the diets induced a malnutrition state, as shown by the good growth performance values [28].

The cell proliferative rate, revealed by *PCNA* staining and gene expression, were higher at AI than at PI in T1, in accordance with the higher cell renewal at the proximate segment of the digestive tract than at the distal one. In fish, under normal conditions, *PCNA* staining is located mainly in the stem cells maintained in the intervillus pockets (equivalent to the Paneth crypts of mammals) of the intestine [58]. SBM-based diets caused an increase in the length of the *PCNA+* proliferative compartment at these pockets [59], which can be reduced by the dietary inclusion of BM or its wall extracts [35, 36]. In the current study, we did not observe the same neat pattern of *PCNA*-reactivity as in Atlantic salmon, as the extreme plant diet (D3) did not induce such an increase in the length of *PCNA+* stretches, or an increase in *PCNA* transcripts. Furthermore, the addition of BP-70 at the highest dose in T1 decreased significantly *PCNA* expression at AI. Therefore, D3 did not induce the typical signs of enteritis as SMB-based diets in salmon, which is indicative of fish species differences in tolerance to high plant inclusion levels.

Electrophysiological techniques with Ussing-type chambers have been used to evaluate intestinal integrity *in vitro* [60], providing a valuable and relatively fast method for the measurement of different electrolytes and substance effects across epithelial tissues from fish to mammals. The current *in vitro* experiments with T3 fish confirmed that the molecular and histological changes induced in fish fed D3 also induced alterations of intestinal physiology, regardless of the fish size, as evidenced by the decrease in Rt. Supplementation with butyrate clearly proved to rescue tissue integrity, as Rt values increased in D4, almost reaching those of D1 fish in small fish (T3-B, fed for 12 months), but not in large fish (T3-A, fed for 38 months). Thus, the higher the differences between D1 and D3, the more difficult to be reversed by D4. Similarly, nucleotide supplementation of high plant protein diets minimized diarrheic signs in meagre (*Argyrosomus regius*) [61]. In addition, differences observed in Rt between large and small control fish can be attributed to the increased thickness of the intestinal muscularis layers with age, as reported in other fish species [62].

In conclusion, extreme plant diets can produce some detrimental effects that butyrate supplementation can restore. Table 4 summarizes the most important changes. In particular, the intestinal inflammatory profile, the antioxidant activation, the imbalance of the genes involved in mucus production, the changes in the epithelial junctions, and the decrease in Rt were returned to the levels of control fish. Recent studies have shown that fish meal replacement also has an impact on fish microbial profile [63]. Thus, further studies are underway to determine the changes induced in the intestinal microbiome and the possible protection of fish against pathogen challenges with this additive.

**Table 4:** Summary of the most important changes induced by the experimental diets in the three gilthead sea bream trials.

TRIAL	DIET	Duration	GROWTH PERFORMANCE	OTHER MAJOR OUTCOMES
1	D4: moderate FM/FO inclusion + 0.8% BP-70 dose	9 wks	No changes	Glycogen accumulation in liver Mucosal foldings increased Gut index increased Leucocyte infiltration in gut Down-regulation of PCNA at AI
2	D3: low FM/FO inclusion	8 mos	No changes^1^	Mild inflammation in gut Up-regulation of pro-inflammatory and antioxidant activation genes Imbalance of mucus related genes Up-regulation of epithelial permeability and structure- related genes
	D4: low FM/FO inclusion + 0.4% BP-70	8 mos	No changes^1^	Abundant goblet cells Recovery of expression profile of defence- and antioxidant-related genes
3	D3: low FM/FO inclusion	12, 38 mos	No changes^2^	Rt decreased
	D4: low FM/FO inclusion + 0.4% BP-70	12, 38 mos	No changes^2^	Rt fully (12 mo) and partially (38) restored

^1^Benedito-Palos et al. 2016 [28];

^2^ Simó-Mirabet et al., 2016 [29].

Rt = Trans-epithelial electrical resistance

## Supporting Information

S1 Table Ingredients and chemical composition of experimental diets fed to gilthead sea bream in trial 1 (T1). (DOCX)

S2 Table Ingredients and chemical composition of experimental diets fed to gilthead sea bream in trial 2 (T2). (DOCX)

S3 Table Forward and reverse primers for real-time PCR of gilthead sea bream genes used in T1 and T2 trials. (DOCX)

S4 Table Gene expression profile of the anterior intestine of gilthead sea bream in trial 2 (T2). Functional gene categories: 1 = cell differentiation and proliferation; 2 = intestinal architecture and permeability; 3 = enterocyte mass and epithelial damage; 4 = interleukins and cytokines; 5 = pattern recognition receptors; 6 = mitochondria function and biogenesis. The experimental diets had different fish meal (FM) and fish oil (FO) contents or supplementation with sodium butyrate (BP-70 ^®^Norel): T2-D1 (FM 25% -FO 15%), T2-D2 (FM 5%—FO 6%), T1-D3 (FM 5%—FO 2.5%) and T1-D4 (FM 5% -FO 2.5%—BP-70 0.4%). *β-actin* was used as a housekeeping gene and all values were referred to the expression level of *ILK* in fish (n = 8) fed the T2-D1 diet. Different superscript letters in the same row indicate significant differences (*P* < 0.05; Student-Newman-Keuls).
(DOCX)

S5 Table Gene expression profile of the posterior intestine of gilthead sea bream in trial 2 (T2). Functional gene categories: 1 = cell differentiation and proliferation; 2 = intestinal architecture and permeability; 3 = enterocyte mass and epithelial damage; 4 = interleukins and cytokines; 5 = pattern recognition receptors; 6 = mitochondria function and biogenesis. The experimental diets had different fish meal (FM) and fish oil (FO) contents or supplementation with sodium butyrate (BP-70 ^®^Norel): T2-D1 (FM 25% -FO 15%), T2-D2 (FM 5%—FO 6%), T1-D3 (FM 5%—FO 2.5%) and T1-D4 (FM 5% -FO 2.5%—BP-70 0.4%). β-actin was used as a housekeeping gene and all values were referred to the expression level of ILK in fish (n = 8) fed the T2-D1 diet. Different superscript letters in the same row indicate significant differences (*P* < 0.05; Student-Newman-Keuls).
(DOCX)

S1 Fig Effect of sodium butyrate (BP-70 ^®^Norel) supplementation in anterior (A, C, E), posterior (B, D) intestine, and liver (F, G) of gilthead sea bream in trial 2 (T2). A, B, F: control diet (T2-D1); C, D, G: extreme plant diet plus BP-70 (T2-D4); E: extreme plant diet (T2-D3). Note the higher number of goblet cells with a different staining pattern in C-D than in A-B, and the lymphocyte-like cell epithelial infiltration in C, D. E: Detail of the lymphocytic infiltration in the epithelial base and the eosinophilic granular cells in T2-D3 intestine. Stainings = Giemsa (A-E), periodic acid-Schiff (F, G). Scale bars = 20 μm.
(TIF)

S2 Fig Representative intestinal sections of gilthead sea bream fed the control diet in trial 1 (T1-D1), comparing anterior (A, C, E) and posterior segments (B, D, F). Stainings: Fatty acid binding protein 2 (A, B); proliferating cell nuclear antigen (C, D); intestinal alkaline phosphatase activity (F, G). Scale bars = 20 μm.
(TIF)

S3 Fig Progression of trans-epithelial electrical resistance (Rt, Ω cm^2^) in the anterior intestine of gilthead sea bream in trial 3 (T3). Fish from T3-A (~1,420 g) and T3-B (~250 g) trials were fed with three different diets (D1: Control; D3: Extreme plant diet; D4; Extreme plant diet plus 0.4% BP-70). Rt was manually recorded at 30 min intervals for 150 min after mounting, and data are presented as mean ± SEM for each time interval.
(DOCX)
