# Supplementary material for: Dietary Butyrate Helps to Restore the Intestinal Status of a Marine Teleost (Sparus aurata) Fed Extreme Diets Low in Fish Meal and Fish Oil
Source: PLoS One. 2016 Nov 29;11(11):e0166564. doi: 10.1371/journal.pone.0166564 (PMC5127657; doi:10.1371/journal.pone.0166564)
Supplement: S1 Table — (DOCX) [file pone.0166564.s001.docx]

**S1 Table.** **Ingredients and chemical composition of experimental diets fed to gilthead sea bream in trial 1 (T1).**

| Ingredient (g/kg) | T1-D1/4 |
| --- | --- |
| Fish meal (CP 70%) ^1^ | 14.9 |
| CPSP 90 ^2^ | 4.9 |
| Corn gluten meal | 39.6 |
| Soybean meal | 14.3 |
| Extruded wheat | 4 |
| Fish oil ^3^ | 10 |
| Rapeseed oil | 0.8 |
| Linseed oil | 2.9 |
| Palm oil | 1.2 |
| Soya lecithin | 1 |
| Binder (sodium alginate) | 1 |
| Mineral premix ^4^ | 1 |
| Vitamin premix ^5^ | 1 |
| CaHPO_4_.2H_2_O (18%P) | 2 |
| L-Lysine | 0.55 |
| Butyrate (BP-70 ®Norel) | 0-0.8% |
| *Proximate composition* |  |
| Protein (% DM) | 48-49 |
| Lipid (% DM) | 22-22.5 |

^1^Fish meal (Scandinavian LT)

^2^Fish soluble protein concentrate (Sopropêche, France)

^3^Fish oil (Sopropêche, France)

^4^Supplied the following (mg / kg diet, except as noted): calcium carbonate (40% Ca) 2.15 g, magnesium hydroxide (60% Mg) 1.24 g, potassium chloride 0.9 g, ferric citrate 0.2 g, potassium iodine 4 mg, sodium chloride 0.4 g, calcium hydrogen phosphate 50 g, copper sulphate 0.3, zinc sulphate 40, cobalt sulphate 2, manganese sulphate 30, sodium selenite 0.3

^5^Supplied the following (mg / kg diet): retinyl acetate 2.58, DL-cholecalciferol 0.037, DL-α tocopheryl acetate 30, menadione sodium bisulphite 2.5, thiamin 7.5, riboflavin 15, pyridoxine 7.5, nicotinic acid 87.5, folic acid 2.5, calcium pantothenate 2.5, vitamin B_12_ 0.025, ascorbic acid 250, inositol 500, biotin 1.25 and choline chloride 500
