# Supplementary material for: Dietary Butyrate Helps to Restore the Intestinal Status of a Marine Teleost (Sparus aurata) Fed Extreme Diets Low in Fish Meal and Fish Oil
Source: PLoS One. 2016 Nov 29;11(11):e0166564. doi: 10.1371/journal.pone.0166564 (PMC5127657; doi:10.1371/journal.pone.0166564)
Supplement: S2 Table — (DOCX) [file pone.0166564.s002.docx]

**S2 Table.** **Ingredients and chemical composition of experimental diets fed to gilthead sea bream in trial 2 (T2).**

| Ingredient (%) | Diet | | | |
| --- | --- | --- | --- | --- |
|  | D1 | D2 | D3 | D4 |
| Fish meal | 23.0 | 3.0 | 3.0 | 3.0 |
| Fish hydrolysate (CPSP) | 2.0 | 2.0 | 2.0 | 2.0 |
| Soya protein | 16.0 | 25.0 | 25.0 | 25.0 |
| Corn gluten | 15.0 | 25.0 | 25.0 | 25.0 |
| Wheat gluten | 4.00 | 7.30 | 7.30 | 7.30 |
| Rapeseed cake | 12.00 | 9.70 | 9.90 | 9.90 |
| Wheat | 11.08 | 6.80 | 6.64 | 6.24 |
| Fish oil | 15.60 | 6.56 | 2.50 | 2.50 |
| Rapeseed oil | 0 | 4.40 | 6.50 | 6.50 |
| Palm olein | 0 | 4.40 | 6.50 | 6.50 |
| Monocalcium phosphate | 0.303 | 2.097 | 2.097 | 2.097 |
| Histidine | 0.136 | 0.136 | 0.136 | 0.136 |
| Mineral-vitamin mix^1^ | 0.500 | 0.500 | 0.500 | 0.500 |
| Cholesterol | 0.113 | 0.113 | 0.113 | 0.113 |
| Amino-acid and micronutrient mix^2^ | 0.20 | 2.92 | 2.74 | 2.74 |
| Antioxidants | 0.045 | 0.045 | 0.045 | 0.045 |
| Yttrium | 0.03 | 0.03 | 0.03 | 0.03 |
| BP-70 | 0 | 0 | 0 | 0.40 |
| *Proximate* *composition* |  |  |  |  |
| Dry matter (DM, %) | 91.65 | 91.79 | 91.80 | 92.34 |
| Crude protein (% DM) | 45.48 | 46.73 | 46.12 | 46.03 |
| Crude fat (% DM) | 19.80 | 19.56 | 20.13 | 19.40 |
| EPA+DHA (% DM) | 2.90 | 1.38 | 0.67 | 0.63 |

^1^Supplied the following (g/kg mix, except as noted): calcium 689, sodium 108, iron 3, manganese 1, zinc 1, cobalt 2 mg, iodine 2 mg, selenium 20 mg, molybdenum 32 mg, retinyl acetate 1, DL-cholecalciferol 2.6, DL-α tocopheryl acetate 28, menadione sodium bisulphite. Ascorbic acid 16, thiamin 0.6, riboflavin 1.7, pyridoxine 1.2, vitamin B12 50 mg, nicotinic acid 5, pantothenic acid 3.6, folic acid 0.6, biotin 50 mg.

^2^ Contains methionine, lysine, choline, lecithin
