# Supplementary material for: Dietary Butyrate Helps to Restore the Intestinal Status of a Marine Teleost (Sparus aurata) Fed Extreme Diets Low in Fish Meal and Fish Oil
Source: PLoS One. 2016 Nov 29;11(11):e0166564. doi: 10.1371/journal.pone.0166564 (PMC5127657; doi:10.1371/journal.pone.0166564)
Supplement: S3 Table — (DOCX) [file pone.0166564.s003.docx]

**S3 Table.** **Forward and reverse primers for real-time PCR of gilthead sea bream genes.**

| Gene name | Symbol |  | Primer sequence |
| --- | --- | --- | --- |
| Proliferating cell nuclear antigen | *PCNA* | F | CGT ATC TGC CGT GAC CTG T |
|  |  | R | AGA ACT TGA CTC CGT CCT TGG |
| Bone morphogenetic protein receptor type-1A | *BMPR1A* | F | AGT GCT GGG CTC ATA ACC |
|  |  | R | CAT CTT GGC GAG TGT CTT CT |
| Indian hedgehog protein | *IHH* | F | ACA GGT TGG CTA TCG CAG TG |
|  |  | R | CCT CCG TCA CAC GCA AGT |
| Zinc finger protein GLI1 | *GLI1* | F | AGA ACC AGC GAG GAA TGC CGT ATT |
|  |  | R | TTG AAG TGG GTC GGT GTC TGT TGA TT |
| Zinc finger protein GLIS3 | *GLIS3* | F | CGA CAG TTG CGG AAG AAG ATG |
|  |  | R | AGG GTG GAT GGT TAA ACA GTC T |
| Hedgehog-interacting protein | *HHIP* | F | CTG TGT AAG AGC GGC TAC T |
|  |  | R | CCT GGT CGT TGG GCA TAC |
| Protein wntless homolog | *WLs* | F | GAG GTC GGC AGC GTG GCT CAT AAG TA |
|  |  | R | GTT GAC AGG CAG ACG GAT GTT GAG AAG GT |
| Transcriptional regulator Myc | *Myc* | F | CAG CAG CAA CCG CAA GTG T |
|  |  | R | TGT CGT AGT CCT CCG TGT CAG A |
| Catenin beta-1 | *CTNNB1* | F | ACA CAG AGA CGC ACC AGC AT |
|  |  | R | CTC CAT ACG AAC TCC CTC CAC AAA |
| Transcription factor 4 | *Tcf4* | F | CAG AGA GCC CAA CCC ACA CT |
|  |  | R | CCC AAC TCG CCA CCC AGT AT |
| Notcheless protein homolog 1 | *NLE1* | F | GGA CTT GAC GAC GGA GAC |
|  |  | R | ACC AGG CGA TGC TCA GTA |
| Transcription factor HES-1-B | *HES1-B* | F | GCC TGC CGA TAT GAT GGA A |
|  |  | R | GGA GTT GTG TTC ATG CTT GC |
| Zinc finger protein GFI-1 | *GFI-1* | F | CTC AGC AGC CTC TGG ACT |
|  |  | R | GCA GTG GTA GGT GTT GGA G |
| Krueppel-like factor 4 | *KLF4* | F | ACA TCA CCG CAC GCA CAC |
|  |  | R | AAC CAC AGC CCT CCC AGT C |
| Integrin beta-1-binding protein 1 | *ITGB1BP1* | F | GCC ACC CTC TCT ACC TGA TAG T |
|  |  | R | TTG AGA GCC AGG AGG TTC TTC |
| Integrin beta-6 | *ITGB6* | F | AGC CTC CCA ACA TCC CTA TGA TTA TTC |
|  |  | R | CTT CCA CAC ACC CAG CAG AA |
| Integrin-linked protein kinase | *ILK* | F | GCC AAT GAA CAC GGG AAC AC |
|  |  | R | ACG AGA TCC TCA GCC ACT TG |
| Occludin | *OCLN* | F | GTG TCA GAA CCT CTA CCA GAC CAG CTA CTC |
|  |  | R | GAA AGC CTC CCA CTC CTC CCA TCT |
| Claudin-3 | *CLDN3* | F | CGC CTT GCC GAT GTG GAG |
|  |  | R | ACC TGA GCA GTG ACG ATG TTG |
| Claudin-12 | *CLDN12* | F | CTC TCA GGG CTA CAC ATC TAC CTA TGC |
|  |  | R | ACA TTC GTG AGC GGC TGG AG |
| Claudin-15 | *CLDN15* | F | CCG ATT GTG GAA GTA GTG GCT CTG GT |
|  |  | R | CAG CAT CAC CCA ACC GAC GAA CC |
| Tight junction protein ZO-1 | *TJP1* | F | AAG CAG TAT TAC GGT GAC TCA |
|  |  | R | TGC ATC CCT GGC TTG TAG |
| Cadherin-1 | *CDH1* | F | TGC TCC ATA CAG CGT CAC CTT ACA |
|  |  | R | CTC GTT CAT CCT AGC CGT CCA GTT |
| Cadherin-17 | *CDH17* | F | GAT GCC CGC AAC CCA GAG |
|  |  | R | CCG TTG ATT CAC TGC CGT AGA C |

**S3 Table.** Continued I

| Gene name | Symbol |  | Primer sequence |  |
| --- | --- | --- | --- | --- |
| Junctional adhesion molecule A | *F11R* | F | GAC TGG TTT CGG TGG CTT TGT TC |  |
|  |  | R | TGG CTT GGG AGG TAG TGA CTG TA |  |
| Coxsackievirus and adenovirus receptor homolog | *CXADR* | F | CAT CAG AGG ACT ACG AGA GG |  |
|  |  | R | CAT CTT GGC AGC ATT TGG T |  |
| Desmoplakin | *DSP* | F | GCA GAA GGA GCA CGA GAC CATC |  |
|  |  | R | GGG TGT TCT TGT CGC AGG TGA A |  |
| Gap junction Cx32.2 protein | *CX32.2* | F | CGA GGT GTT CTA TCT GCT CTG TA |  |
|  |  | R | CTT GTG GGT GCG AGT CCT |  |
| Gap junction Cx32.7 protein | *CX32.7* | F | CGC TCA CCT TGC CCT CAC AA |  |
|  |  | R | AAC CAG ATG ATG ACC GAC TTC TCT |  |
| Gap junction beta-4 protein | *GJB4* | F | TGA AAT CCT CTA CCT GGT CGG CAA AC |  |
|  |  | R | TGG CGA GAA TTA TGG AAC GAG GTG AAG |  |
| Mucin 2 | *MUC2* | F | ACG CTT CAG CAA TCG CAC CAT |  |
|  |  | R | CCA CAA CCA CAC TCC TCC ACA T |  |
| Mucin 2-like | *MUC2-like* | F | GTG TGT GGC TGT GTT CCT TGC TTT GT |  |
|  |  | R | GCG AAC CAG TCT GGC TTG GAC ATC A |  |
| Mucin 13 | *MUC13* | F | TTC AAA CCC GTG TGG TCC AG |  |
|  |  | R | GCA CAA GCA GAC ATA GTT CGG ATA T |  |
| Intestinal mucin | *I-MUC* | F | GTG TGA CCT CTT CCG TTA |  |
|  |  | R | GCA ATG ACA GCA ATG ACA |  |
| Intestinal-type alkaline phosphatase | *ALPI* | F | CCG CTA TGA GTT GGA CCG TGA T |  |
|  |  | R | GCT TTC TCC ACC ATC TCA GTA AGG G |  |
| Liver type fatty acid-binding protein | *FABP1* | F | GTC CTC GTC AAC ACC TTC ACC AT |  |
|  |  | R | CGC CTT CAT CTT CTC GCC AGT |  |
| Intestinal fatty acid-binding protein | *FABP2* | F | CGA GCA CAT TCC GCA CCA AAG |  |
|  |  | R | CCC ACG CAC CCG AGA CTT C |  |
| Ileal fatty acid-binding prote | *FABP6* | F | ACC CAG GAC GGC AAT ACC |  |
|  |  | R | CGA CGG TGA AGT TGT TGG T |  |
| Calreticulin | *CALR* | F | GGC GGC GGC TAT GTG AAG |  |
|  |  | R | GCA TCG CAG TCT GAT CCA AGT C |  |
| Calnexin | *CANX* | F | CCC GAG GGT TGG CTA GAT GA |  |
|  |  | R | GGC GTC TGG GTC TCC GAT AT |  |
| Glutathione reductase | *GR* | F | TGT TCA GCC ACC CAC CCA TCG G |  |
|  |  | R | GCG TGA TAC ATC GGA GTG AAT GAA GTC TTG |  |
| Glutathione S-transferase 3 | *GST3* | F | CCA GAT GAT CAG TAC GTG AAG ACC GTC |  |
|  |  | R | CTG CTG ATG TGA GGA ATG TAC CGT AAC |  |
| Superoxide dismutase [Cu-Zn] | *SOD1* | F | TCA CGG ACA AGA TGC TCA CTC TC |  |
|  |  | R | GGT TCT GCC AAT GAT GGA CAA GG |  |
| Interleukin 1 beta | *IL-1β* | F | GCGACCTACCTGCCACCTACACC |  |
|  |  | R | TCG TCC ACC GCC TCC AGA TGC |  |
| Interleukin 1 receptor type 1 | *IL-1R1* | F | GAA GCT GTA CGA CGC CTA C |  |
|  |  | R | CTC CAC TGC CTT ACT GTA TCC |  |
| Interleukin 6 | *IL-6* | F | TCT TGA AGG TGG TGC TGG AAG TG |  |
|  |  | R | AAG GAC AAT CTG CTG GAA GTG AGG |  |
| Interleukin 6 receptor subunit beta | *IL-6RB* | F | CAG TGT CGG AGT ATG TGG TTG AGT | |
|  |  | R | CCC TCT GCC AGT CTG TCC AA | |
| Interleukin 7 | *IL-7* | F | CTA TCT CTG TCC CTG TCC TGT GA | |
|  |  | R | TGC GGA TGG TTG CCT TGT AAT | |
| Interleukin 15 | *IL-15* | F | GAG ACC AGC GAG CGA AAG GCA TCC | |
|  |  | R | GCC AGA ACA GGT TAC AGG TTG ACA GGA A | |
| Interleukin 8 | *IL-8* | F | CAG CAG AGT CTT CAT CGT CAC TAT TG | |
|  |  | R | AGG CTC GCT TCA CTG ATG G | |
| High affinity interleukin-8 receptor A | *IL-8RA* | F | CTT GTT TCA TCT GAC GAT AG | |
|  |  | R | AAG AGG ATG CTT GTG TAG | |

**S3 Table.** Continued II

| Gene name | Symbol |  | Primer sequence | |
| --- | --- | --- | --- | --- |
| Interleukin 10 | *IL-10* | F | AAC ATC CTG GGC TTC TAT CTG | |
|  |  | R | GTG TCC TCC GTC TCA TCT G | |
| Interleukin10 receptor subunit alpha | *IL-10RA* | F | GAG GAC AAT GAA GAG GAA GAC AGG AG | |
|  |  | R | TGT TCG TAG CGG AGT TGG ACT | |
| Interleukin 34 | *IL-34* | F | TCT GTC TGC CTG CTG GTA G | |
|  |  | R | ATG CTG GCT GGT GTC TGG | |
| Tumor necrosis factor alpha | *TNFα* | F | CAGGCGTCGTTCAGAGTCTC | |
|  |  | R | CTGTGGCTGAGAGGTGTGAG | |
| Complement C1q tumour necrosis factor 3 | *CTRP3* | F | ATG CTG TGC TGA GAG AGA TGA G | |
|  |  | R | AGT CTT CTG CTT CTC CTG CTC | |
| Macrophage colony-stimulating factor 1 receptor 1 | *CSF1R1* | F | TTG CGT GTG GTG AGG AAG GAA GGT | |
|  |  | R | AGC AGG CAG GGC AGC AGG TA | |
|  |  |  |  | |
| C-X-C motif chemokine 9 | *CXCL9* | F | CTG AGG AGT AAC GAG ACA GTG TG | |
|  |  | R | CCT GTT CCA GCA GCG TAT CA | |
| C-C motif chemokine 21 | *CCL21* | F | GCA ACA TCC CTG CCA CAA TCT TCA | |
|  |  | R | TCC TTC AGT TCT ATG ACC CAC ATC TCT C | |
| C-C chemokine receptor type 3 | *CCR3* | F | CTA CAT CAG CAT CAC CAT ACG CAT CCT | |
|  |  | R | TGG CAC GGC ACT TCT CCT TCA | |
| C-C chemokine receptor type 9 | *CCR9* | F | TCC CTG AGT TAA TCT TCG CCC AAG TG | |
|  |  | R | TGT TGT ATT CGT TGT TCC AGT AGA CCA GAG | |
| C-C chemokine receptor type 11 | *CCR11* | F | GCT ACG ATT ACA GTT ATG AA | |
|  |  | R | TAG ATG ATT GGG AGG AAG | |
| C-C chemokine CK8 | *CK8* | F | CCG TCC TCA TCT GCT TCA TAC T | |
|  |  | R | GCT CTG CCG TTG ATG GAA C | |
| CD48 antigen | *CD48* | F | GAC ATA CTT CGA GGT TGG CGG TAA ACT | |
|  |  | R | GAT GTT GTC GAT AGT CTC CGT CAC TGT AGG | |
| CD276 antigen | *CD276* | F | GTC ACA CTC AAC TGC TCC TTC A | |
|  |  | R | CGC CAG AAG ACG GTC AGA T | |
| Toll-like receptor 1 | *TLR1* | F | GGG ACC TGC CAG TGT GTA AC | |
|  |  | R | GCG TGG ATA GAG TTG GAC TTG AG | |
| Toll-like receptor 2 | *TLR2* | F | CAT CTG CGA CTC TCC TCT CTT CCT | |
|  |  | R | ATT CAA CAA TGG AGC GGT GGA CTT | |
| Toll-like receptor 5 | *TLR5* | F | TCG CCA ATC TGA CGG ACC TGA G | |
|  |  | R | CAG AAC GCC GAT GTG GTT GTA AGA C | |
| Toll-like receptor 9 | *TLR9* | F | GCC TTC CTT GTC TGC TCT TTC T | |
|  |  | R | GCC GTA GAG GTG CTT CAG TAG | |
| Nucleotide-bindingprotein oligomerization domain-containing protein 1 | *NOD1* | F | GTC CAG GTT GAG GAG CAT CCA GTG |  |
|  |  | R | TGA AGC CAC AAG CCG ACA GGT T |  |
|  |  |  |  |  |
| Macrophage mannose receptor 1 | *MRC1* | F | CTT CCG ACC GTA CCT GTA CCT ACT CA |  |
|  |  | R | CGA TTC CAG CCT TCC GCA CAC TTA |  |
| CD209 antigen | *CD209* | F | CGC CAC GAG CAT GAG GAC AA |  |
|  |  | R | TCT TGC CAG AAT CCA TCA CCA TCC A |  |
| CD302 antigen | *CD302* | F | GGA CCA GAG GAA GAG CAC ATC |  |
|  |  | R | GAC CAG GGC GGA CAT CAG |  |
| C-type lectin domain family 10 member A | *CLEC10A* | F | CGA CTC TGG ACT CCC TCA |  |
|  |  | R | CGT TGT TGA TGG TGC GTT C |  |
| Galectin-1 | *LGALS1* | F | GTG TGA GGA GGT CCG TGA TG |  |
|  |  | R | ACT GTA GAG CCG TCC GAT AGG |  |
| Galectin-8 | *LGALS8* | F | GGC GGT GAA CGG CGG TCA |  |
|  |  | R | GCT CCA GCT CCA GTC TGT GTT GAT AC |  |
| **S3 Table.** Continued III |  |  |  |  |
| Gene name | Symbol |  | Primer sequence | |
| L-rhamnose-binding lectin CSL2 | *CSL2* | F | GCT CAC CAA TAC AAA GTG CTC TCA G |  |
|  |  | R | CTT GCC ATC ACA CCT CCT CCT |  |
| Fucolectin | *FCL* | F | CCA TAC TGC TGA ACA GAC CAA CC |  |
|  |  | R | TGA TGG AGG TGA CGA TGT AGG A |  |
| Vimentin | *VIM* | F | GCT TCA GAC AGG ATG TGG ACA AC |  |
|  |  | R | AGT GAT TCT ACC TTC CGC TCC AG |  |
| Mitochondrial 10 kDa heat shock protein | *mtHsp10* | F | CAT GCT GCC AGA GAA GTC TCA AGG |  |
|  |  | R | AGG TCC CAC TGC CAC TAC TGT |  |
| Mitochondrial 60 kDa heat shock protein | *mtHsp60* | F | TGT GGC TGA GGA TGT GGA TGG AGA G |  |
|  |  | R | GCC TGT TGA GAA CCA AGG TGC TGA G |  |
| Mitochondrial 70 kDa heat shock protein | *mtHsp70* | F | TCC GGT GTG GAT CTG ACC AAA GAC |  |
|  |  | R | TGT TTA GGC CCA GAA GCA TCC ATG |  |
| Enoyl-CoA hydratase | *ECH* | F | GCC CAA GAA GCC AAG CAA TCA G |  |
|  |  | R | CTT TAG CCA TAG CAG AGA CCA GTT TG |  |
| Hydroxyacyl-CoA dehydrogenase | *HADH* | F | GAA CCT CAG CAA CAA GCC AAG AG |  |
|  |  | R | CTA AGA GGC GGT TGA CAA TGA ATC C |  |
| Citrate synthase | *CS* | F | TCC AGG AGG TGA CGA GCC |  |
|  |  | R | GTG ACC AGC AGC CAG AAG AG |  |
| Mitochondrial import inner membrane translocase subunit 44 | *Tim44* | F | GAT GAC CTG GGA CAC ACT GG |  |
|  |  | R | TCA CTC CTC TTC CTG AGT CTG G |  |
| Mitochondrial import receptor subunit Tom22 | *Tom22* | F | CGC TCT GGG TGG GTA CTA CCT CCT T |  |
|  |  | R | CGA ACA CAA CAG GCA GCA CCA GGA T |  |
| Mitochondrial Transcription factor A | *mtTFA* | F | GAG CCC GCA ACA GAA ACA GCC ATT |  |
|  |  | R | ACT GCT CCC TGT CCC GCT GAT AG |  |
| Nuclear respiratory factor 1 | *NRF1* | F | CAG ATA GTC CTG GCA GAG A |  |
|  |  | R | GAC CTG TGG CAT CTT GAA |  |
| Proliferator-activated receptor gamma coactivator 1 alpha | *PGC1α* | F | CGT GGG ACA GGT GTA ACC AGG ACT C |  |
|  |  | R | ACC AAC CAA GGC AGC ACA CTC TAA TTC T |  |
| β-actin | *ACTB* | F | TCC TGC GGA ATC CAT GAG A |  |
|  |  | R | GAC GTC GCA CTT CAT GAT GCT |  |
