# Supplementary material for: Dietary Butyrate Helps to Restore the Intestinal Status of a Marine Teleost (Sparus aurata) Fed Extreme Diets Low in Fish Meal and Fish Oil
Source: PLoS One. 2016 Nov 29;11(11):e0166564. doi: 10.1371/journal.pone.0166564 (PMC5127657; doi:10.1371/journal.pone.0166564)
Supplement: S4 Table — Functional gene categories: 1 = cell differentiation and proliferation; 2 = intestinal architecture and permeability; 3 = enterocyte mass and epithelial damage; 4 = interleukins and cytokines; 5 = pattern recognition receptors; 6 = mitochondria function and biogenesis. The experimental diets had different fish meal (FM) and fish oil (FO) contents or supplementation with sodium butyrate (BP-70 ®Norel): T2-D1 (FM 25% -FO 15%), T2-D2 (FM 5%—FO 6%), T1-D3 (FM 5%—FO 2.5%) and T1-D4 (FM 5% -FO 2.5%—BP-70 0.4%). β-actin was used as a housekeeping gene and all values were referred to the expression level of ILK in fish (n = 8) fed the T2-D1 diet. Different superscript letters in the same row indicate significant differences (P < 0.05; Student-Newman-Keuls). (DOCX) [file pone.0166564.s004.docx]

**S4 Table. Gene expression profile of the anterior intestine of gilthead sea bream in trial 2 (T2).** Functional gene categories: 1 = cell differentiation and proliferation; 2 = intestinal architecture and permeability; 3 = enterocyte mass and epithelial damage; 4 = interleukins and cytokines; 5 = pattern recognition receptors; 6 = mitochondria function and biogenesis.

| Functional | Gene | T2-D1 | |  | T2-D2 | |  | T2-D3 | |  | T2-D4 | |  | *ANOVA* |
| --- | --- | --- | --- | --- | --- | --- | --- | --- | --- | --- | --- | --- | --- | --- |
| category |  | Mean | SEM |  | Mean | SEM |  | Mean | SEM |  | Mean | SEM |  | *P-value* |
| 1 | *PCNA* | 1.69^a^ | 0.11 |  | 1.57^ab^ | 0.24 |  | 1.24^b^ | 0.04 |  | 1.74^a^ | 0.23 |  | 0.050 |
|  | *HES1-B* | 0.83^a^ | 0.07 |  | 0.86^a^ | 0.03 |  | 1.20^b^ | 0.13 |  | 0.72^a^ | 0.08 |  | 0.001 |
|  | *KLF4* | 0.59^a^ | 0.06 |  | 0.84^b^ | 0.03 |  | 0.84^b^ | 0.07 |  | 0.85^b^ | 0.07 |  | 0.027 |
|  | *BMPR1A* | 0.59 | 0.04 |  | 0.54 | 0.03 |  | 0.65 | 0.08 |  | 0.58 | 0.04 |  | 0.224 |
|  | *GLI1* | 0.10 | 0.01 |  | 0.09 | 0.01 |  | 0.09 | 0.01 |  | 0.09 | 0.01 |  | 0.320 |
|  | *HHIP* | 0.54 | 0.05 |  | 0.52 | 0.05 |  | 0.55 | 0.03 |  | 0.50 | 0.05 |  | 0.159 |
|  | *WLs* | 0.17 | 0.01 |  | 0.16 | 0.01 |  | 0.16 | 0.01 |  | 0.15 | 0.01 |  | 0.250 |
|  | *Myc* | 0.09 | 0.01 |  | 0.08 | 0.01 |  | 0.08 | 0.01 |  | 0.08 | 0.01 |  | 0.743 |
|  | *CTNNB1* | 3.45 | 0.24 |  | 2.93 | 0.11 |  | 3.38 | 0.32 |  | 3.14 | 0.18 |  | 0.205 |
| 2 | *OCLN* | 2.09^ab^ | 0.14 |  | 1.92^a^ | 0.10 |  | 2.90^b^ | 0.52 |  | 1.79^a^ | 0.12 |  | 0.040 |
|  | *CDH1* | 5.76^a^ | 0.37 |  | 4.73^a^ | 0.26 |  | 7.39^b^ | 1.36 |  | 5.97^ab^ | 0.19 |  | 0.025 |
|  | *CX32.2* | 19.6^a^ | 1.64 |  | 26.8^b^ | 2.14 |  | 23.6^ab^ | 2.33 |  | 18.2^a^ | 1.83 |  | 0.027 |
|  | *MUC2* | 19.6^a^ | 2.44 |  | 21.4^ab^ | 2.43 |  | 30.5^b^ | 3.76 |  | 21.8^ab^ | 1.82 |  | 0.050 |
|  | *MUC13* | 31.2^a^ | 3.22 |  | 28.5^a^ | 1.93 |  | 45.8^b^ | 5.91 |  | 25.1^a^ | 2.39 |  | 0.001 |
|  | *ILK* | 1.00 | 0.06 |  | 0.87 | 0.04 |  | 1.04 | 0.06 |  | 0.93 | 0.05 |  | 0.022 |
|  | *CDH17* | 22.02 | 0.79 |  | 20.25 | 0.51 |  | 19.61 | 1.04 |  | 21.3 | 0.83 |  | 0.136 |
|  | *CXADR* | 1.80 | 0.10 |  | 1.60 | 0.05 |  | 1.98 | 0.25 |  | 1.67 | 0.08 |  | 0.081 |
| 3 | *GR* | 1.97^ab^ | 0.30 |  | 1.88^a^ | 0.08 |  | 2.44^b^ | 0.22 |  | 1.97^a^ | 0.07 |  | 0.006 |
|  | *PRDX1* | 13.2^ab^ | 1.94 |  | 10.5^a^ | 1.57 |  | 17.9^b^ | 3.49 |  | 9.67^a^ | 1.51 |  | 0.050 |
|  | *ALPI* | 19.70 | 1.92 |  | 17.38 | 0.99 |  | 22.08 | 2.73 |  | 19.9 | 2.64 |  | 0.556 |
|  | *FABP2* | 91.19 | 10.62 |  | 95.76 | 8.72 |  | 77.02 | 13.6 |  | 60.7 | 12.1 |  | 0.176 |
|  | *CALR* | 8.89 | 0.90 |  | 9.10 | 1.23 |  | 12.49 | 2.93 |  | 8.70 | 0.71 |  | 0.278 |
| 4 | *IL-6* | 0.008^a^ | 0.002 |  | 0.010^ab^ | 0.001 |  | 0.015^b^ | 0.003 |  | 0.009^ab^ | 0.001 |  | 0.050 |
|  | *IL-12B* | 0.14^a^ | 0.01 |  | 0.14^a^ | 0.01 |  | 0.20^b^ | 0.02 |  | 0.16^ab^ | 0.01 |  | 0.042 |
|  | *CCR11* | 2.17^a^ | 0.21 |  | 2.21^ab^ | 0.11 |  | 2.85^b^ | 0.32 |  | 1.89^a^ | 0.19 |  | 0.033 |
|  | *IL-6RB* | 0.68 | 0.07 |  | 0.55 | 0.05 |  | 0.064 | 0.051 |  | 0.53 | 0.06 |  | 0.285 |
|  | *IL-8* | 0.15 | 0.03 |  | 0.12 | 0.01 |  | 0.15 | 0.03 |  | 0.14 | 0.02 |  | 0.254 |
|  | *IL-8RA* | 0.06 | 0.01 |  | 0.04 | 0.01 |  | 0.07 | 0.01 |  | 0.03 | 0.01 |  | 0.456 |
|  | *IL-10* | 0.08 | 0.01 |  | 0.05 | 0.01 |  | 0.09 | 0.01 |  | 0.06 | 0.01 |  | 0.118 |
|  | *IL-10RA* | 0.30 | 0.03 |  | 0.23 | 0.03 |  | 0.33 | 0.02 |  | 0.29 | 0.02 |  | 0.110 |
|  | *TNFα* | 0.05 | 0.01 |  | 0.05 | 0.01 |  | 0.06 | 0.01 |  | 0.06 | 0.00 |  | 0.192 |
|  | *CXC* | 2.45 | 0.24 |  | 2.33 | 0.21 |  | 2.34 | 0.42 |  | 2.71 | 0.34 |  | 0.833 |
|  | *CCR3* | 0.40 | 0.04 |  | 0.35 | 0.04 |  | 0.44 | 0.04 |  | 0.36 | 0.05 |  | 0.630 |
| 5 | *MRC1* | 0.39^b^ | 0.05 |  | 0.28^ab^ | 0.01 |  | 0.28^ab^ | 0.02 |  | 0.25^a^ | 0.03 |  | 0.133 |
|  | *LGALS1* | 1.43^a^ | 0.17 |  | 1.55^ab^ | 0.24 |  | 2.12^b^ | 0.49 |  | 1.28^a^ | 0.19 |  | 0.074 |
|  | *LGALS8* | 0.98^a^ | 0.08 |  | 0.94^a^ | 0.03 |  | 1.30^b^ | 0.16 |  | 0.80^a^ | 0.05 |  | 0.001 |
|  | *CSL2* | 1.95^a^ | 0.50 |  | 0.12^a^ | 0.05 |  | 6.11^b^ | 2.4 |  | 0.88^a^ | 0.52 |  | 0.030 |
|  | *TLR1* | 0.33 | 0.03 |  | 0.28 | 0.02 |  | 0.33 | 0.03 |  | 0.28 | 0.02 |  | 0.195 |
|  | *CLEC10A* | 0.01 | 0.00 |  | 0.02 | 0.00 |  | 0.01 | 0.001 |  | 0.01 | 0.00 |  | 0.441 |
|  | *FCL* | 27.2 | 5.02 |  | 20.4 | 3.06 |  | 34.2 | 13.3 |  | 16.6 | 3.13 |  | 0.333 |
| 6 | *mtHsp10* | 1.74 | 0.10 |  | 2.19 | 0.32 |  | 2.01 | 0.19 |  | 2.27 | 0.22 |  | 0.203 |
|  | *mtHsp70* | 1.41 | 0.07 |  | 1.15 | 0.07 |  | 1.64 | 0.32 |  | 1.52 | 0.10 |  | 0.114 |
