# Supplementary material for: Dietary Butyrate Helps to Restore the Intestinal Status of a Marine Teleost (Sparus aurata) Fed Extreme Diets Low in Fish Meal and Fish Oil
Source: PLoS One. 2016 Nov 29;11(11):e0166564. doi: 10.1371/journal.pone.0166564 (PMC5127657; doi:10.1371/journal.pone.0166564)
Supplement: S5 Table — Functional gene categories: 1 = cell differentiation and proliferation; 2 = intestinal architecture and permeability; 3 = enterocyte mass and epithelial damage; 4 = interleukins and cytokines; 5 = pattern recognition receptors; 6 = mitochondria function and biogenesis. The experimental diets had different fish meal (FM) and fish oil (FO) contents or supplementation with sodium butyrate (BP-70 ®Norel): T2-D1 (FM 25% -FO 15%), T2-D2 (FM 5%—FO 6%), T1-D3 (FM 5%—FO 2.5%) and T1-D4 (FM 5% -FO 2.5%—BP-70 0.4%). β-actin was used as a housekeeping gene and all values were referred to the expression level of ILK in fish (n = 8) fed the T2-D1 diet. Different superscript letters in the same row indicate significant differences (P < 0.05; Student-Newman-Keuls). (DOCX) [file pone.0166564.s005.docx]

**S5 Table.** **Gene expression profile of the posterior intestine of gilthead sea bream in trial 2 (T2).** Functional gene categories: 1 = cell differentiation and proliferation; 2 = intestinal architecture and permeability; 3 = enterocyte mass and epithelial damage; 4 = interleukins and cytokines; 5 = pattern recognition receptors.

| Functional | Gene | T2-D1 | |  | T2-D2 | |  | T2-D3 | |  | T2-D4 | |  | *ANOVA* |
| --- | --- | --- | --- | --- | --- | --- | --- | --- | --- | --- | --- | --- | --- | --- |
| category |  | Mean | SEM |  | Mean | SEM |  | Mean | SEM |  | Mean | SEM |  | *P-value* |
| 1 | *HHIP* | 0.77^ab^ | 0.04 |  | 0.90^b^ | 0.06 |  | 0.73^ab^ | 0.07 |  | 0.61^a^ | 0.06 |  | 0.023 |
|  | *Myc* | 0.14^ab^ | 0.03 |  | 0.17^b^ | 0.03 |  | 0.08^a^ | 0.01 |  | 0.10^a^ | 0.02 |  | 0.049 |
|  | *HES1-B* | 1.12^ab^ | 0.07 |  | 1.16^b^ | 0.02 |  | 0.87^a^ | 0.10 |  | 0.86^a^ | 0.07 |  | 0.026 |
|  | *PCNA* | 0.96 | 0.11 |  | 0.95 | 0.08 |  | 0.79 | 0.10 |  | 0.88 | 0.10 |  | 0.593 |
|  | *BMPR1A* | 0.58 | 0.05 |  | 0.60 | 0.04 |  | 0.52 | 0.03 |  | 0.50 | 0.04 |  | 0.274 |
|  | *GLI1* | 0.11 | 0.01 |  | 0.12 | 0.01 |  | 0.12 | 0.02 |  | 0.08 | 0.01 |  | 0.190 |
|  | *WLs* | 0.31 | 0.02 |  | 0.35 | 0.02 |  | 0.30 | 0.04 |  | 0.26 | 0.03 |  | 0.177 |
|  | *CTNNB1* | 3.04 | 0.21 |  | 2.83 | 0.10 |  | 2.87 | 0.22 |  | 2.86 | 0.13 |  | 0.799 |
|  | *KLF4* | 0.58 | 0.06 |  | 1.07 | 0.22 |  | 0.98 | 0.24 |  | 1.15 | 0.23 |  | 0.213 |
| 2 | *DSP* | 3.82^a^ | 0.21 |  | 3.9^a^ | 0.17 |  | 3.03^b^ | 0.26 |  | 3.41^ab^ | 0.18 |  | 0.025 |
|  | *ILK* | 1.04 | 0.07 |  | 1.12 | 0.04 |  | 1.02 | 0.09 |  | 0.94 | 0.07 |  | 0.354 |
|  | *OCLN* | 2.15 | 0.19 |  | 2.16 | 0.14 |  | 2.10 | 0.19 |  | 2.00 | 0.15 |  | 0.897 |
|  | *CDH1* | 5.32 | 0.27 |  | 4.92 | 0.23 |  | 4.43 | 0.38 |  | 4.73 | 0.38 |  | 0.278 |
|  | *CDH17* | 15.1 | 1.39 |  | 11.5 | 0.98 |  | 12.1 | 1.04 |  | 13.86 | 0.50 |  | 0.070 |
|  | *MUC2* | 15.3 | 1.30 |  | 16.3 | 1.57 |  | 16.2 | 1.33 |  | 18.2 | 2.88 |  | 0.558 |
|  | *MUC13* | 24.2 | 2.19 |  | 19.4 | 1.73 |  | 26.7 | 3.29 |  | 22.4 | 1.75 |  | 0.181 |
|  | *I-MUC* | 3.70 | 2.17 |  | 4.52 | 1.94 |  | 0.38 | 0.17 |  | 0.34 | 0.18 |  | 0.127 |
| 3 | *ALPI* | 6.12 | 1.07 |  | 5.57 | 0.84 |  | 6.81 | 0.72 |  | 6.58 | 0.51 |  | 0.341 |
|  | *FABP2* | 195.1 | 48.2 |  | 140.6 | 44.6 |  | 108.9 | 24.1 |  | 147.6 | 24.5 |  | 0.518 |
|  | *FABP6* | 354.7 | 45.5 |  | 474.9 | 75.5 |  | 464.1 | 91.9 |  | 523.3 | 99.7 |  | 0.125 |
|  | *CALR* | 8.43 | 1.18 |  | 10.03 | 0.87 |  | 8.22 | 1.02 |  | 7.90 | 0.67 |  | 0.410 |
|  | *GR* | 1.56 | 0.09 |  | 1.25 | 0.08 |  | 1.38 | 0.11 |  | 1.35 | 0.07 |  | 0.095 |
|  | *PRDX1* | 7.00 | 0.84 |  | 6.96 | 1.05 |  | 8.26 | 2.28 |  | 5.93 | 0.52 |  | 0.243 |
| 4 | *IL-6* | 0.007 | 0.00 |  | 0.009 | 0.00 |  | 0.009 | 0.00 |  | 0.008 | 0.00 |  | 0.584 |
|  | *IL-6RB* | 0.46 | 0.05 |  | 0.51 | 0.04 |  | 0.57 | 0.05 |  | 0.49 | 0.07 |  | 0.470 |
|  | *IL-8* | 0.21 | 0.06 |  | 0.21 | 0.03 |  | 0.23 | 0.04 |  | 0.19 | 0.05 |  | 0.966 |
|  | *IL-8RA* | 0.03 | 0.00 |  | 0.04 | 0.01 |  | 0.04 | 0.01 |  | 0.04 | 0.01 |  | 0.734 |
|  | *IL-10* | 0.09 | 0.02 |  | 0.07 | 0.01 |  | 0.08 | 0.01 |  | 0.07 | 0.01 |  | 0.710 |
|  | *IL-10RA* | 0.27 | 0.04 |  | 0.24 | 0.01 |  | 0.25 | 0.01 |  | 0.27 | 0.03 |  | 0.847 |
|  | *IL-12B* | 0.16 | 0.02 |  | 0.14 | 0.02 |  | 0.15 | 0.02 |  | 0.14 | 0.02 |  | 0.913 |
|  | *TNFα* | 0.06 | 5.65 |  | 0.06 | 7.23 |  | 0.06 | 0.00 |  | 0.06 | 0.01 |  | 0.807 |
|  | *CXC* | 4.21 | 0.54 |  | 4.13 | 0.37 |  | 2.83 | 0.31 |  | 4.16 | 0.59 |  | 0.128 |
|  | *CCR3* | 0.46 | 0.06 |  | 0.48 | 0.05 |  | 0.45 | 0.05 |  | 0.39 | 0.05 |  | 0.671 |
|  | *CCR11* | 1.77 | 0.12 |  | 1.63 | 0.16 |  | 2.08 | 0.28 |  | 1.69 | 0.15 |  | 0.108 |
|  | *CK8* | 8.20 | 1.14 |  | 8.27 | 1.23 |  | 8.77 | 1.19 |  | 6.18 | 1.28 |  | 0.454 |
| 5 | *MRC1* | 0.45^a^ | 0.04 |  | 0.42^a^ | 0.03 |  | 0.39^ab^ | 0.06 |  | 0.28^b^ | 0.03 |  | 0.023 |
|  | *TLR1* | 0.28 | 0.03 |  | 0.26 | 0.02 |  | 0.25 | 0.01 |  | 0.26 | 0.03 |  | 0.833 |
|  | *CLEC10A* | 0.022 | 0.003 |  | 0.026 | 0.003 |  | 0.023 | 0.003 |  | 0.013 | 0.003 |  | 0.144 |
|  | *LGALS1* | 3.10 | 0.21 |  | 3.93 | 0.39 |  | 3.11 | 0.40 |  | 2.71 | 0.44 |  | 0.062 |
|  | *LGALS8* | 1.02 | 0.13 |  | 1.23 | 0.17 |  | 0.94 | 0.10 |  | 0.83 | 0.10 |  | 0.185 |
|  | *CSL2* | 6.51 | 1.88 |  | 5.45 | 1.25 |  | 7.01 | 1.61 |  | 4.08 | 1.07 |  | 0.200 |
|  | *FCL* | 26.7 | 6.25 |  | 23.9 | 4.65 |  | 31.5 | 4.46 |  | 14.1 | 2.81 |  | 0.058 |
