# Supplementary figures and images for: Dietary Butyrate Helps to Restore the Intestinal Status of a Marine Teleost (Sparus aurata) Fed Extreme Diets Low in Fish Meal and Fish Oil
Source: PLoS One. 2016 Nov 29;11(11):e0166564. doi: 10.1371/journal.pone.0166564 (PMC5127657; doi:10.1371/journal.pone.0166564)

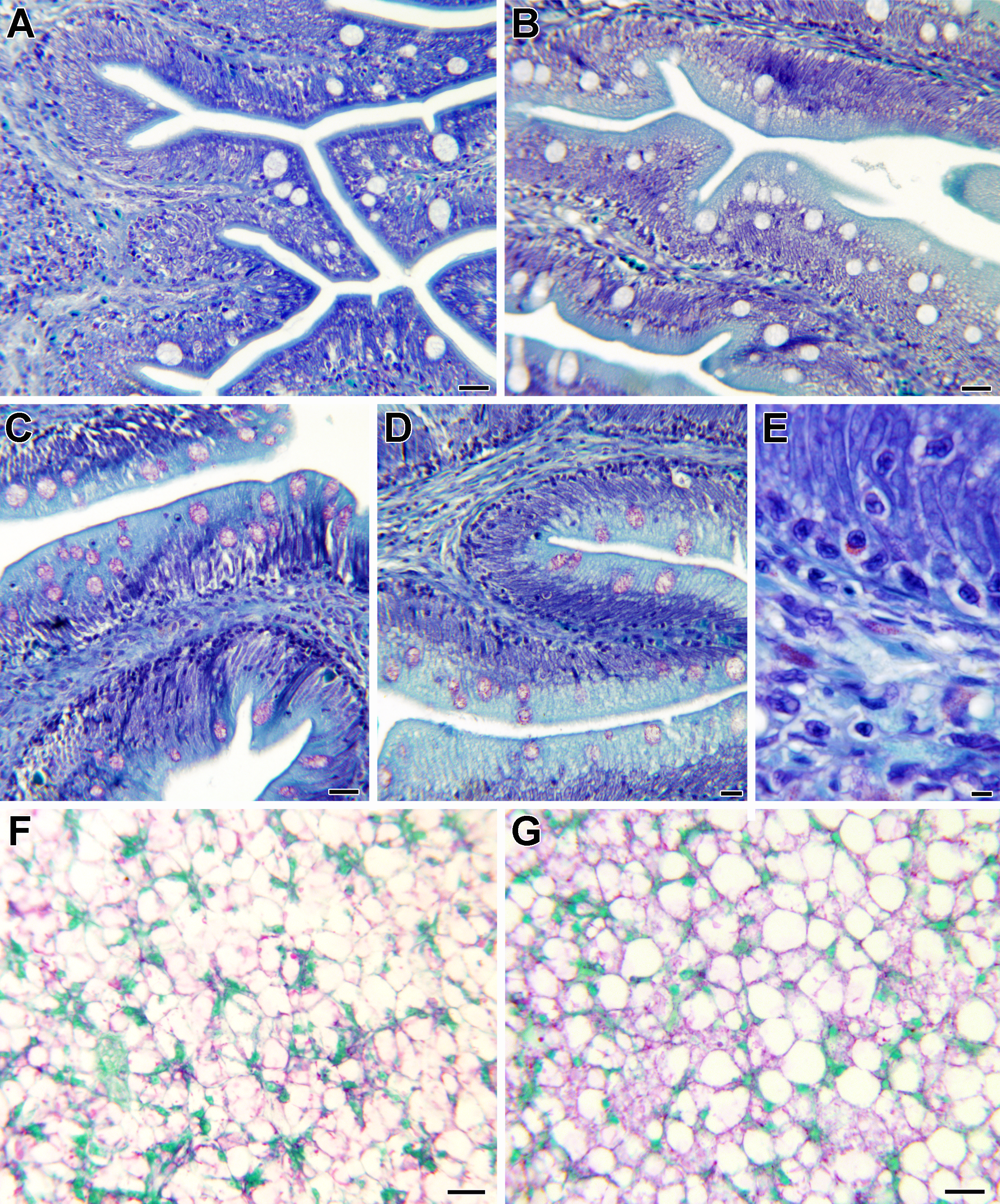

Supplement: S1 Fig — A, B, F: control diet (T2-D1); C, D, G: extreme plant diet plus BP-70 (T2-D4); E: extreme plant diet (T2-D3). Note the higher number of goblet cells with a different staining pattern in C-D than in A-B, and the lymphocyte-like cell epithelial infiltration in C, D. E: Detail of the lymphocytic infiltration in the epithelial base and the eosinophilic granular cells in T2-D3 intestine. Stainings = Giemsa (A-E), periodic acid-Schiff (F, G). Scale bars = 20 μm. (TIF) [file pone.0166564.s006.tif]

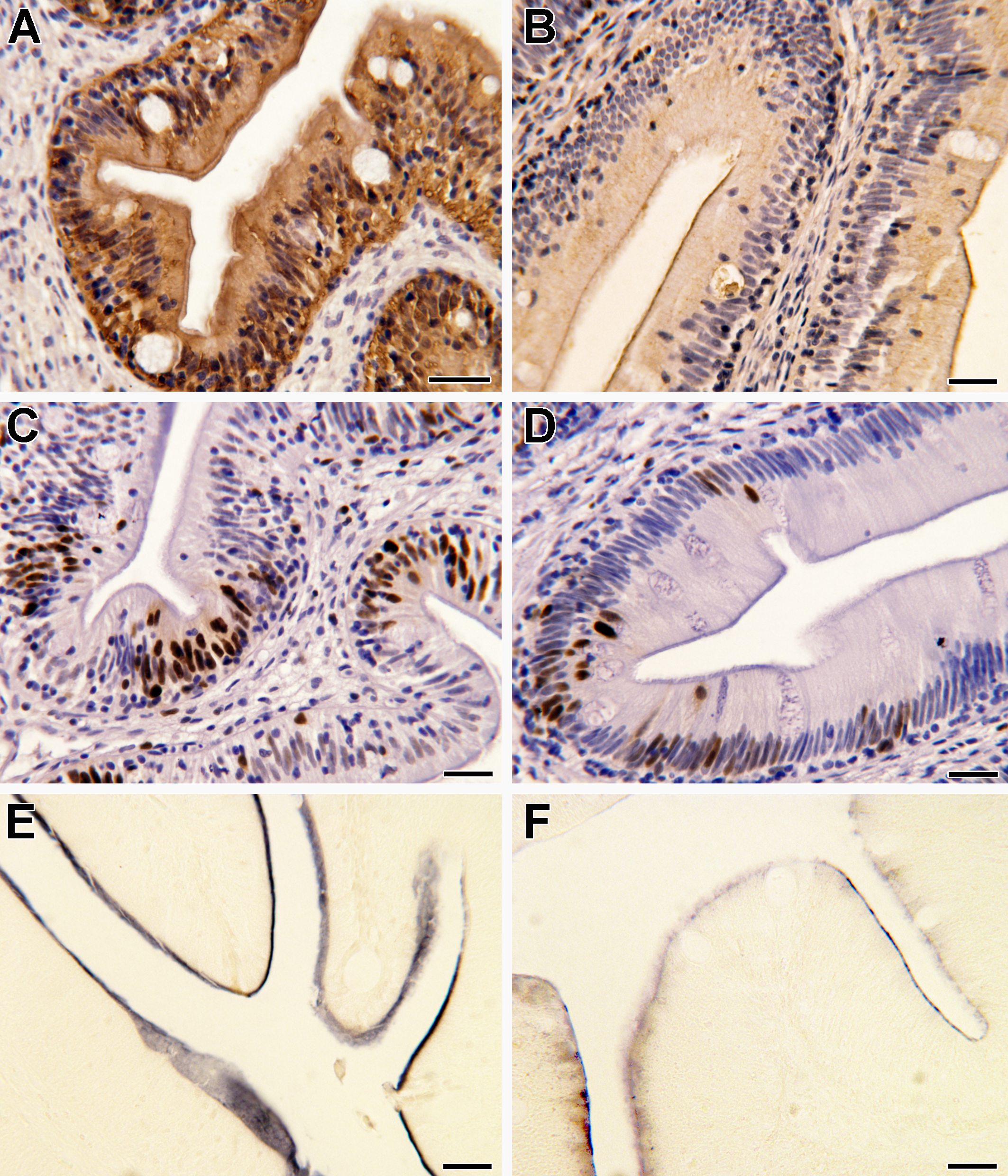

Supplement: S2 Fig — Stainings: Fatty acid binding protein 2 (A, B); proliferating cell nuclear antigen (C, D); intestinal alkaline phosphatase activity (F, G). Scale bars = 20 μm. (TIF) [file pone.0166564.s007.tif]
