# Supplementary material for: Dietary Butyrate Helps to Restore the Intestinal Status of a Marine Teleost (Sparus aurata) Fed Extreme Diets Low in Fish Meal and Fish Oil
Source: PLoS One. 2016 Nov 29;11(11):e0166564. doi: 10.1371/journal.pone.0166564 (PMC5127657; doi:10.1371/journal.pone.0166564)
Supplement: S3 Fig — Fish from T3-A (~1,420 g) and T3-B (~250 g) trials were fed with three different diets (D1: Control; D3: Extreme plant diet; D4; Extreme plant diet plus 0.4% BP-70). Rt was manually recorded at 30 min intervals for 150 min after mounting, and data are presented as mean ± SEM for each time interval. (DOCX) [file pone.0166564.s008.docx]

**S3 Fig**. **Progression of trans-epithelial electrical resistance (Rt, Ω cm^2^) in the anterior intestine of gilthead sea bream in trial 3 (T3).**
